# Defensive healthcare practice: systematic review of qualitative evidence

**DOI:** 10.1136/bmjopen-2024-085673

**Published:** 2024-07-18

**Authors:** Theo Lorenc, Claire Khouja, Melissa Harden, Helen Fulbright, James Thomas

**Affiliations:** 1 Centre for Reviews and Dissemination, University of York, York, UK; 2 University College London Social Science Research Unit, London, UK

**Keywords:** systematic review, qualitative research, medical law, physicians, clinical decision-making

## Abstract

**Objective:**

To synthesise qualitative evidence on clinicians’ views and experiences of defensive practice.

**Design:**

Systematic review of qualitative data.

**Data sources:**

MEDLINE, Embase, PsycINFO, AMED, Maternity and Infant Care, CINAHL, ASSIA, Sociological Abstracts, Proquest Dissertations & Theses and PROSPERO were searched from 2000 to October 2023.

**Eligibility criteria:**

We included English-language studies of clinicians which reported qualitative data on the impact of litigation or complaints on clinical practice.

**Data extraction and synthesis:**

We coded findings data line by line using a grounded theory approach. We assessed quality using Hawker *et al*’s tool and synthesised data thematically.

**Results:**

17 studies were included. Participants identify a range of clinical decisions which may be defensively motivated, relating to diagnosis and documentation as well as to treatment. Defensive practice often relates to a diffuse sense of risk rather than the direct threat of litigation and may overlap with other motivations, such as perceived pressure from patients or the desire to avoid harm. Defensive practice is seen to be harmful in many ways, but again, these perceptions may gain force from broader narratives of mistrust and disempowerment, as much as from the risk of litigation.

**Conclusions:**

The idea of defensive practice, as enacted, is more complex than some theoretical accounts suggest and may often function to express broader concerns about the work of clinical care. The qualitative evidence calls into question the view of defensive practice as a key mediator linking litigation risk to inappropriate treatment and excess costs.

**STRENGTHS AND LIMITATIONS OF THIS STUDY**

- This is a comprehensive systematic review of the qualitative evidence from clinicians on defensive practice.
- The review focuses on the impact of litigation and complaints on clinical practice.
- There are some limitations in the evidence base, such as possible selection bias.
- Patients’ and other stakeholders’ views were not included.

## Introduction

 The term ‘defensive medicine’ refers to clinicians’ modifying their practice to reduce the likelihood of litigation or complaints as a result of negative outcomes. This may include a range of practices, including both overtreatment (eg, ordering unnecessary diagnostic tests or treatments) and undertreatment (eg, avoiding risky but potentially beneficial treatments or avoiding high-risk patients).^1^ Defensive practice may have negative consequences for patient care, both directly due to the impacts of inappropriate treatment and indirectly by undermining care quality (eg, antibiotic stewardship)^2^ and fostering unrealistic norms of clinical practice (so-called ‘gold plating’, where practices adopted defensively become incorporated into routine care).^3 4^ Defensive practice has also widely been seen as a potential driver of excess healthcare costs, due to the additional costs of carrying out tests or procedures.

Survey studies have found that many clinicians report practising defensive medicine, with many studies finding rates of 80%–90% or even higher.^5^,^9^ More sophisticated quantitative research has sought to understand the correlates of defensive practice by investigating associations between the determinants of defensive practice (eg, self-reported or objectively measured litigation risk) with putative impacts (eg, total healthcare costs).^10^,^12^ However, this research generally leaves unclear how these perceptions play out in reality, and how defensive decision-making becomes established in clinical practice. Qualitative research may help to fill in the picture outlined by the quantitative data, by situating concerns about litigation or other relevant factors in the day-to-day realities of clinical practice.

There are also challenges in defining and operationalising the idea of defensive medicine. The fear of litigation or complaints is likely to interact with other factors determining clinical decision-making, and it is arguably impossible to objectively quantify the relative contributions of these factors. If defensive medicine is defined in opposition to clinically optimal care, there may be an irreducible element of judgement in deciding between the two in practice. Survey studies find that reports of defensive medicine may vary significantly depending on the framing of the question.^13^ Hence, it is challenging to interpret the quantitative evidence in terms of causal drivers of defensive practice, and a critical attitude to these data seems warranted.^14^ Both the true costs of defensive practice, and the extent to which it is driven specifically by litigation risk rather than by broader dynamics, remain somewhat unclear.^4^

There is also a question as to the applicability of these findings. Much of the quantitative research and the broader thinking about defensive practice originates in the USA, and may not be generalisable to countries with less market-led healthcare systems. There are several potential differences here. The market for malpractice insurance in the USA, where many clinicians purchase cover as individuals, is quite different to that in other settings such as the UK where this is less common.^15^ Concern about increased malpractice litigation and spiralling insurance costs, which have formed the background of thinking around defensive medicine in the USA,^16^,^18^ have been less of a focus in other countries, and concerns about defensive medicine in the latter may relate to broader consequences such as patient complaints.^8^ Hence, while self-reported rates of defensive practice appear to be high across countries, it is unclear how far the overarching theoretical narrative developed from the US data can be applied more generally. The broader context of clinicians’ work—the incentive structures provided by different reimbursement systems, or the cultural norms which influence patients’ expectations—also vary internationally in ways which may have an impact on defensive decision-making.

It is thus important to consider questions about defensive medicine within the broader context of clinical practice, in order to gain a more nuanced understanding of both the impacts which defensive medicine may have on patient outcomes and the healthcare system, and the potential value of systemic policy change in improving suboptimal care (as well as to investigate the situation in countries other than the USA). Qualitative evidence may help to illuminate these broader questions. One previous review includes qualitative evidence on clinicians other than doctors^1^; however, that review does not cover evidence on doctors and, while systematic in principle, it is limited in its searching and synthesis. Two recent scoping reviews cover relevant studies but do not carry out qualitative synthesis.^19 20^ Our review aimed to synthesise qualitative evidence on defensive practice from all professional groups of clinicians.

## Methods

This review was conducted in accordance with the CRD Methods Manual^21^ and reported in accordance with PRISMA guidelines. Data were managed using EPPI-Reviewer Web software. The review protocol was registered on PROSPERO before work commenced (registration number CRD42020166559).

The review question is: What is known from qualitative studies about how clinicians modify their practice due to the fear of litigation, complaints, criminal prosecution and/or professional regulation?

### Searching

The search strategies are shown in online supplemental appendix A. The search strategy was designed in Ovid MEDLINE using a range of subject headings and free-text terms relating to the practice of defensive medicine, including clinicians’ fear of legal or disciplinary action. A search filter was incorporated into the strategy to restrict retrieval to qualitative studies.^22^ Further qualitative terms were added to the search filter to increase sensitivity, including terms to capture any qualitative reviews or mixed-methods studies. A date limit was applied to restrict retrieval to studies published from 2000 onwards. The searches were not restricted by language. The following sources were initially searched in January 2020, with the search updated in October 2023.

- MEDLINE ALL (Ovid).
- Embase (Ovid).
- PsycINFO (Ovid).
- Allied and Complementary Medicine—AMED (Ovid).
- Maternity and Infant Care (Ovid).
- Cumulative Index to Nursing & Allied Health—CINAHL Complete (Ebsco).
- Applied Social Science Index and Abstracts—ASSIA (ProQuest).
- Sociological Abstracts (ProQuest).
- ProQuest Dissertations & Theses A&I (ProQuest).
- PROSPERO.

In addition, the reference lists of included studies and relevant systematic reviews were scanned; forward citation chasing was carried out using Web of Science; the websites of relevant organisations were handsearched and Google Scholar was searched using a simplified version of the MEDLINE search strategy and the first 50 hits screened.

### Screening

The inclusion criteria were as follows:

1. Does the study report qualitative data?
2. Does the study report data collected from clinicians?
3. Does the study mainly focus on litigation or complaints?
4. Does the study mainly focus on perceptions of the impact of litigation or complaints on clinical practice?
5. Is the study published in English?

We focused on studies of clinicians (rather than patients or other stakeholders) partly for pragmatic reasons, in order to make the review manageable, and partly because we anticipated that patients would be less likely to frame views about care in terms of the impact of litigation (we return to this point as a potential limitation in the discussion below). An initial sample of 10% of abstracts was screened independently by two reviewers and differences resolved by discussion. Agreement at this stage was judged to be adequate, and the remaining abstracts were screened by a single reviewer. The full text of every reference meeting the criteria, or where it was unclear whether it met the criteria, was reviewed and rescreened against the same criteria by two reviewers independently.

### Quality assessment, data extraction and synthesis

We assessed the quality of included studies using Hawker *et al*’s tool.^23^ This tool provides a structured instrument to evaluate quality across several methodological domains, including sampling, data collection and analysis, ethics and bias, and transferability. Studies were not excluded nor downgraded based on quality assessment rating, but information on study quality was used informally to guide the synthesis. We extracted contextual data on the studies including information on the study methods, the sample and the setting. We coded qualitative data line by line using a grounded theory approach. Quality assessment and contextual data extraction were carried out by one reviewer and checked in detail by another; thematic coding was carried out by one reviewer and the analysis reviewed by another. The structure of the themes is shown in table 1. The overarching framework consisted of three domains: definitions and understandings of defensive practice; causal factors influencing defensive practice and the impacts of defensive practice across a range of domains. More specific codes were developed within this structure as shown in table 1. Coding was inductive, with new codes developed in discussion between the two reviewers undertaking the analysis; where new codes were added, all data were reread to identify data corresponding to the code. Development of the coding structure was guided by thematic saturation.

**Table 1:** Thematic structure

What is defensive practice?	General views
	Treatment
	Testing and monitoring
	Documentation
Motivations for defensive practice	Litigation and complaints
	Relations with clinical peers
	Patient factors
	Social factors
	Adverse patient events
Impacts of defensive practice	Professionalism and autonomy
	Impacts on patient care
	Communication and relationships
	Adherence to guidelines
	Emotional impacts

### Patient and public involvement

We did not include any patient and public involvement for this project.

## Findings

**Figure 1:**
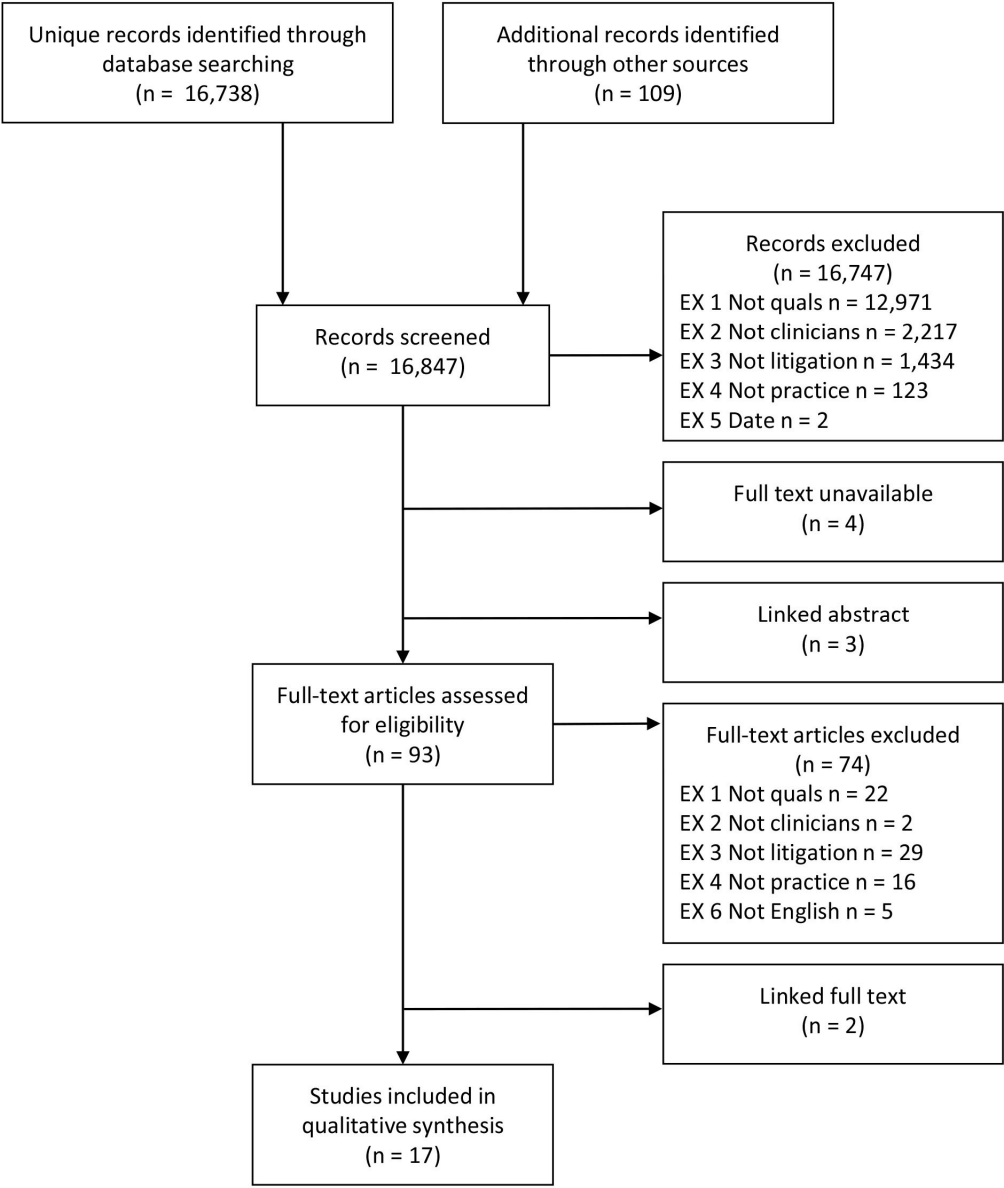
Flow of literature through the review.

The flow of literature through the review is shown in figure 1. A total of 16 738 unique records were located by the database searches; supplementary searches provided an additional 109 records. After screening, 17 studies (19 study reports) were included in the review (studies excluded at full-text stage are listed in online supplemental appendix B).

The results of quality assessment are shown in online supplemental appendix C. Overall, the quality of the included studies was moderate to good; however, there were weaknesses across the evidence base in sampling (question 4) and transferability (question 8). Contextual information about the studies is shown in table 2. The most commonly studied area of clinical practice was obstetrics or midwifery (n=10). Clinical populations included doctors (n=11), midwives (n=7) and nurses (n=2). A range of countries was represented, with the greatest number (n=7) from the UK and only one from the USA. This contrasts with the quantitative literature, which as noted above has been dominated by data from the USA.

**Table 2:** Studies included in the review

Short ID	Country	Setting	Population	Sample size	Study focus
Assing Hvidt *et al*^24 51^	Denmark	Primary care	Doctors	28	Perceptions of defensive practice
Bradder^25^	England+Wales	Obstetrics/midwifery	Doctors	50	Risk and litigation in clinical practice
Broom *et al*^40^	Australia	Hospital	Doctors, pharmacists, nurses	29	Influences on antibiotic use
Cunningham and Dovey^35^	New Zealand	Hospital	Doctors	12	Defensive practice and complaints experience
Eftekhari *et al*^26^	Iran	Various	Doctors	43	Views of defensive practice
Hammer^27^	Switzerland	Obstetrics/midwifery	Doctors	26	Risk of malpractice claims
Hindley and Thomson^36^	England	Obstetrics/midwifery	Midwives	58	Defensive practice as motivation for use of fetal intrapartum monitoring
Hood *et al*^28^	Australia	Obstetrics/midwifery	Midwives	16	Views of regulation/litigation
Manuel and Crowe^29^	New Zealand	Mental health	Nurses	10	Clinical responsibility/accountability
Papadopoulos *et al*^30^	USA	Obstetrics/midwifery	Doctors	15	Threat of malpractice litigation
Ries *et al*^31^	Australia	Various	Doctors	17	Views of defensive practice and low-value care
Robertson and Thomson^37^	England	Obstetrics/midwifery	Midwives	22	Litigation impact on practice
Ruston^39^	UK	Primary care	Doctors	85	Referral decisions for patients with breast problems
Spendlove^32^	England	Obstetrics/midwifery	Doctors, midwives	37	Experiences of risk
Surtees^33^	New Zealand	Obstetrics/midwifery	Midwives	40	Risk and impact on practice
Symon^34 52^	Scotland+England	Obstetrics/midwifery	Midwives, doctors	23	Views of defensive practice
Wier^38^	England	Obstetrics/midwifery	Midwives	20	Professional regulation

The synthesis of findings is reported below under three broad headings: (1) general understandings of defensive medicine; (2) motivations and influences on defensive practice and (3) perceived impacts of defensive practice.

### What is defensive medicine?

#### General views

Many participants expressed a view that defensive practice was highly prevalent both in general and in their own individual practice.^24^,^34^ Participants described a pervasive ethos of ‘conservative’ or ‘butt-covering’ decisions, where the possibility of litigation strongly influenced treatment decisions.^28 29^

On the other hand, some participants strongly rejected the idea that fear of litigation influenced their practice, arguing that they were motivated only by the interests of patients.^25 27^ Participants in one study regarded the idea of defensive medicine as a ‘scarecrow’ (participant)^27^ rather than an accurate description of their experience. This diversity of views may relate to the point that defensive motivations largely emerge in the ‘grey area’ (participant)^25^ of clinical judgement, where there is room for legitimate disagreement about the clinically best course of action. What one person regards as defensive practice, another might see as appropriate caution. Thus, whether a given decision is regarded as primarily defensive may be a matter of judgement and context, and the abstract ‘common knowledge’ (participant)^33^ that defensive practice is widespread may not be reflected in how clinicians understand specific cases.

#### Treatment

Despite widespread assent to the general narrative of defensive practice, relatively few participants mentioned specific treatment decisions. The most commonly mentioned example was caesarean section.^25 27 32 34 35^ Other than this specific area, few clinicians identified specific treatment practices which they felt frequently represented defensive practice. Two study authors remarked that participants either identified few specific examples, or examples which were arguable when discussed in more depth,^25 27^ even though many participants in these studies were familiar with the concept of defensive medicine and clearly endorsed the broader narrative behind the term.

Even in the case of caesarean section, participants held nuanced views, noting that defensive concerns may be present ‘in the back of one’s mind’^34^ even where the treatment decision is clinically justifiable. There is thus an irreducible ambiguity in many cases of defensive practice because clinical judgement cannot be eliminated in favour of a purely objective account of the reasons behind a decision.^34^

You could argue the baby is ill. To subject them to the stress of induction of labour is illogical. To give them an elective caesarean section is a planned procedure, with all the best people there, is the better decision. In real life there’s always a grey area in the middle where you can't make up your mind. (participant)^25^

#### Testing and monitoring

More participants mentioned the overuse of diagnostic tests or monitoring as an aspect of defensive practice.^24^,^2628 31^ Clinicians may order tests or examinations that they know are unlikely to provide clinically useful information, but where there is an outside possibility of identifying serious problems. However, in most cases, this seems to be motivated less by the fear of litigation or complaint than by a fear of adverse outcomes.

[I]t’s to do with what you do know, and what you don't know, and you would be scared of not doing various investigations in case you miss something that you didn't think you were looking for in the first place. (participant)^25^

Again, the most specific and detailed data come from the obstetric context, where participants drew a clear link between fear of litigation and the overuse of electronic fetal monitoring (EFM).^28 32 33 36 37^ Some participants linked the overuse of EFM to a more general medicalisation of birth, and a perception that care is driven by technology rather than the best interests of the patient.^28 32^

I’m sure we do EFM because they are always on about litigation, and you know if you don’t get it checked or don’t document it, then if something did go wrong … but I mean that’s very defensive kind of practice isn’t it? (participant)^36^

#### Documentation

The most commonly mentioned change in practice across all the studies was an increase in documentation.^2425 27 29^,^38^ Looking across the evidence, it appears that excessive documentation, rather than decisions about treatment or diagnosis, was the primary example of defensive practice; Bradder notes that the theme of documentation was ‘[o]ne exception to the general lack of consensus over defensive strategies’ (author).^25^ Participants’ remarks on documentation revealed a strong sense of a pervasive practice driven by a generalised fear of litigation, such that all decisions must be constantly recorded and justified. They felt that clinicians’ roles were distorted by an overemphasis on ‘correct’ documentation at the expense of care, particularly emotionally supportive care.^27 32 34 37 38^

I mean all your documentation is all tied up with litigation, the whole lot. You write screeds and screeds to cover yourself. All the time covering, covering, covering … whether it’s right or not. (participant)^33^

However, fear of litigation was not the only factor driving defensive overdocumentation. Guidance and targets produce an increasing demand for paperwork to assess performance or provide feedback.^24 32^ Documentation is thus overdetermined as a theme, suggesting how clinicians’ understanding of defensive practice encompasses both the fear of specific negative events and the broader practical pressures of institutional or regulatory settings.

Another recurring theme when reflecting on own experiences with DM was the demand to document (what some of the GPs described as ‘limitless, meaningless documentation’) that the government policy had imposed on the GPs for quality appraisal purposes. One practice that was particularly described as defensive by the GPs was the documentation of patient records involving long enumerations of negative clinical findings. (author)^24^

### Motivations for defensive medicine

#### Litigation and complaints

The most commonly mentioned motivation for defensive medicine was the fear of being sued, being subject to formal complaints, or being suspended or struck off by professional regulators.^24^,^2832 33 36 39^

One ends up referring some patients with a sort of medico-legal fear behind the scene because you know if you go and reassure someone, and then she turns up in six months time with a carcinoma it’s not going to look good in court. So there is some medico-legal pressures on us to refer some patients to the breast clinic. (participant)^39^

Defensive practice… that is what it’s all about, we don’t practice how we feel we should… midwives are toeing the line because they are frightened of losing their registration… and that’s your livelihood isn’t it? (participant)^38^

However, relatively few participants referred to their own actual experience of being subject to lawsuits or complaints. ‘It must be stressed that risk of malpractice claims as a true cause for concern in our sample was seldom referred to precise facts, concrete events or experiences’ (authors).^27^ Rather, the fear of litigation was diffuse and may have been related to hearsay about what happens to others more than to first-hand experience, with one participant distinguishing a broader ‘indirect’ impact of litigation risk as potentially more important than the ‘direct’ impact.^25^ Assing Hvidt *et al*’s study reported that ‘[e]very GP had experienced being either a subject or co-subject of a patient complaint at some stage in their career’ (authors)^24^ but also suggested that this was not a major driver of changes in practice, perhaps because most of these complaints were felt to be ‘unjustified or ridiculous’ (authors).^24^ Thus, the experience of complaints was not a sufficient condition for practising defensively. Rather, clinicians’ reactions to the risk of complaints or litigation were influenced by a range of broader factors.

#### Relations with clinical peers

One important influence was the views of professional peers. The fear of litigation appears to have more impact where clinicians felt that they are not supported by their institutions or their professional peers. The sudden loss of support, and the sense of being ‘on your own’, was a key focus of fear in these narratives.^2431^,^33^ Complaints which professional peers regarded as well founded—or have themselves initiated—were a more salient object of fear than those which are agreed by peers to be frivolous.

I think because of the blame culture, we’re frightened to do our own job, because if you don’t do it perfectly and something happens, you’re going to be sued and … your name’s going to be dragged through the mud … Sometimes you feel a little bit isolated, so you don’t trust the people that are there allegedly to support you. (participant)^32^

More generally, motivations for defensive medicine are bound up with relationships among colleagues, particularly between junior and senior clinicians.^25^ Clinicians reported involving colleagues, particularly more senior colleagues, as a way to mitigate risk.^25 26 29 37 38^ Decisions to admit or refer patients may also be a driver of defensive practice. In some cases, specific services may have criteria governing admission, for example, regarding diagnostic tests. Referrals may also be driven by regulations or clinical guidelines, sometimes against the referring clinician’s own judgement.^24^ This may sometimes reflect a lack of confidence or experience among junior staff (or at least this perception by senior staff),^25^ although participants in one study suggested that newly qualified clinicians were less likely to practise defensively as they had not had experience of litigation or adverse outcomes.^28^

#### Patient factors

Some participants also mentioned pressure from patients or families as a driver of defensive practice.^24^,^2831 35 36 39 40^ Several participants reported stereotypes about which patients are likely to be ‘demanding’, based for example on socioeconomic or occupational groups such as teachers or lawyers.^24 25 27^ In other cases, these judgements were more based on individual characteristics such as patients’ personalities.^26 33 39^ At the individual clinician level, communication with patients may be an important factor in avoiding the need for defensive practices.^27 31^

Participants in one study suggested that there was considerable nuance in these decisions.^39^ Some described cases of referrals which were straightforwardly defensive, in that they were mainly driven by patient pressure and threats of litigation while others suggested that patient anxiety may be a legitimate reason for a referral, regardless of whether the clinician feels it is well founded. While the two kinds of cases are experienced very differently by clinicians, they may not be easy to distinguish in practice. As with previous themes, reasonable caution—manifest here as a concern for patients’ mental well-being as well as their physical health—may not be clearly distinguished from the fear of litigation.

#### Social factors

Some participants linked pressure from patients to a broader culture of ‘consumerism’ and patients’ willingness to challenge medical authority.^24 26^ They also suggested that broader social norms have shifted in the direction of lowered tolerance of risk and higher expectations of treatment outcomes, which make defensive practice more necessary^24 27 33^; media coverage of medical issues may contribute to this.^24 27^ This change in norms was seen to reinforce a ‘blame culture’ where negative events are seen not as acceptable risks but as outcomes of a mistake by an identifiable individual.^27 32 37^

We’re in that society, that culture at the moment where somebody is always looking to blame … there’s always a scapegoat … and it’s sad that it’s like that … risk is dictating everything we do in maternity. (participant)^32^

Relatively few participants identified specific aspects of the legislative or policy context as potential drivers of defensive practice. In two studies, one from Denmark^24^ and one from Australia^40^ participants argued that private health insurance may be a motivator. In one study from Switzerland, participants identified lower levels of compensation awards and regulation on legal fees as limiting clinicians’ litigation risk, and hence reducing defensive practice.^27^ Some participants saw litigation risk as primarily a problem with the US healthcare system and defensive practice as a symptom of ‘Americanisation’^27 33^ (as we only located one study from the USA, which presented fairly limited relevant data, we cannot directly compare perceptions).

#### Adverse patient events

However, as noted above, these concerns often had less to do with litigation or complaints specifically, than with a fear of adverse patient events for their own sake.^24 25 39 40^ This suggests that defensive practice is bound up with a broader sense of caution in clinical decision-making (although this is not to say that this caution is not itself excessive in some cases).

[Participant 1] Just overlooking something that has disastrous consequences for another human being—it does not even have to elicit a complaint, but just the risk of overlooking something, I mean that is terrible!

[Participant 2] Yes, then I’d rather play it safe.

[Participant 1] Yes, but this has nothing to do with the complaints! (participants)^24^

### Impacts of defensive medicine

#### Professionalism and autonomy

A frequent theme across the studies was that defensive practice undermined clinicians’ professional judgement and autonomy, reduced job satisfaction and increased stress and the likelihood of burn-out, due to the dissonance involved in making decisions which are thought to be clinically suboptimal.^24 25 27 28 30 32 35 37 38 40^ However, participants mostly linked this theme not to litigation risk but rather to the burden of administrative protocols and clinical guidelines. Initiatives aiming to improve accountability and care quality were widely seen as calling clinical authority into question, and as symptomatic of a reduced respect for clinicians’ role and professional judgement.^24 25 27^

Along with the bureaucratic burden, a growing body of legal rules and recommendations bearing on medical work was felt to be a latent societal distrust towards professional skills and their ability to act in the patient’s best interest: “Ultimately, this is what has changed, society does no longer trust doctor’s common sense.” (author/participant)^27^

Midwives, in particular, linked the theme of reduced autonomy and an erosion of their professional role to a more general dynamic of medicalisation. Defensive practice was seen to involve a shift of clinical authority from midwives to obstetricians. Midwives described ongoing conflict and a sense of disempowerment which left them feeling ‘frustrated’ and ‘exhausted’,^28 32^ driven not just by individual relationships but by institutional and professional regulation.

We don’t practice as autonomously as we used to, and there’s a lot of doctor input now into management of women because of litigation, that’s my perception. (participant)^28^

Well I do wonder if it [risk] will affect professional roles more, as changing roles is going on already now. We’re already in litigation ages, we are so aware of litigation and the risks in childbirth … and we are becoming more defensive because of it … it is bound to affect role boundaries in some way. (participant)^32^

#### Impacts on patient care

Participants also argued that defensive practice had negative impacts on patient care, both because of the inherent harms of overtreatment and overdiagnosis^2428 31^,^35^ and because of the diversion of clinician time into formal procedures and away from patient care.^27 32 37 38^

We spend more time with administrative things than really taking care of patients, just to justify and prove that we do our job properly. (participant)^27^

Several participants noted that defensive concerns had made them more likely to avoid certain patients or specialisms,^28 30 33 35 37 38^ particularly patients in challenging situations or with complex needs who were seen to be risky.^35 38^ Some participants reported avoiding rural or less well-equipped clinical settings for similar reasons.^33 35^ Midwives in Hood *et al*’s study mentioned a wide repertoire of strategies to reduce risk, including focusing on antenatal or postnatal care rather than delivery, moving to administrative or research roles, moving to night shifts and avoiding managerial responsibilities.^28^

#### Communication and relationships

More broadly, clinician–patient relationships were felt to suffer because adversarial concerns about litigation risk get in the way of engaging empathetically with patients’ needs.^24 30^ Participants emphasised trust as the core of an effective clinician–patient relationship and argued that defensive practice undermined this trust. Midwives emphasised that their role as advocates for women was dependent on a trusting relationship which was more difficult to maintain under the threat of liability.^28 37^

[My relationship with patients changed] from one of focusing on caring for them physically and emotionally, to always seeing them as a potential adversary. (participant)^30^

Again, for some participants, the direct risk of litigation seemed to be less important than the policy responses to risk. Consent forms, for example, were described by participants in one study as ‘a dehumanisation of the contact you have with your clients, with people who trust you’ (participant).^27^ Defensive practice may also take the form of greater adherence to guidelines, which some participants described as a ‘safety net’ reducing anxiety about litigation.^28^ At the same time, participants in several studies saw guideline-driven treatment as opposed to individualised, responsive care and as leading to overtreatment.^2833^,^35^

To avoid that, you become more mechanistic, more stuck to protocol—you’re also less likely to establish a therapeutic relationship. (participant)^35^

[Litigation] probably means you're practising more defensively, where before you could treat people as individuals and adapt your practice to suit the individual … now there maybe is a tendency to control from a policy document. (participant)^34^

On the other hand, some positive views of the impacts on communication were also expressed. A few participants mentioned that attending to the risk of litigation or complaints had improved communication with patients, for example, by encouraging reflection on practice or going to greater lengths to inform patients and secure consent.^34 35^

#### Emotional impacts

Finally, participants described negative emotional impacts as a result of defensive practice. Making decisions they felt were not clinically well-grounded led to feelings of guilt and regret. More broadly, while defensive decision-making may be motivated by avoiding anxiety, the culture of defensive practice itself was seen to produce chronic anxiety and frustration.^24 25 28 30 32 37^

## Discussion

This review finds that many clinicians agreed that clinical decision-making is at least sometimes influenced by the fear of litigation or complaints, and that this can lead to suboptimal care. However, the studies found few concrete examples, other than caesarean section, of how this impacts treatment; overdiagnosis and overdocumentation were often cited as examples of defensive practice. Several participants suggested that defensive motivations may coexist and interact with other clinically legitimate motives, and that deciding which is primary may be more a matter of clinical judgement than an objective fact.

Many participants saw the threat of litigation as pervasive and unavoidable, even where the objective risk was low, and perceived it as particularly threatening when they felt isolated from their professional peers. However, other motivations also entered into defensive decision-making: the desire to avoid adverse events; pressure from patients or families; the loss of trust in the clinician–patient relationship; and a broader culture which was seen to be intolerant of risk and suspicious of clinicians in general.

The strengths of this review include the systematic methodology, with a clearly defined question enabling the minimisation of bias in the selection of literature and sensitive search strategies. The well-defined question may also be a limitation, in that we excluded potentially illuminating data. For example, we excluded studies of clinicians’ experiences of the litigation or complaint process if they did not also examine the impact of these on practice, and general studies of overtreatment if they did not focus on defensive practice. There may be some limitations in the primary data, particularly selection bias leading to a focus on participants with a particular angle on the question. The findings cover a range of settings and participants, and there may be limitations to transferability; in particular, studies of childbirth appear to raise somewhat different themes than those of other settings. The large body of data on caesarean sections and EFM suggests that litigation risk may play an outsized role in obstetrics and midwifery; this may be due to the potentially severe and lifelong consequences of medical error in childbirth, and hence the potential for large compensation awards following litigation. Another substantial limitation is that we only included studies of clinicians, not patients. Some relevant data from non-clinician populations are available,^41^ and there may also be relevant insights in qualitative literature on related topics such as overdiagnosis^42^; our findings should be seen in this broader context.

The findings suggest that clinicians broadly accept the narrative of defensive practice in the policy literature, which posits a linear path from litigation risk to suboptimal treatment, and thence to increased costs and poorer patient outcomes. However, a more detailed analysis of their own reported experiences and views calls this narrative into question in two ways. First, much defensive practice takes place in the ‘grey area’ of clinical judgement, where concern about litigation may be present but plausibly deniable. Reasonable caution resulting from the fear of adverse events may be impossible to separate from the fear of lawsuits or complaints resulting from those adverse events, and the former appears to often be uppermost in clinicians’ minds. Whether or not a given decision is identified as an example of defensive practice, then, may have less to do with the specific factors entering into the decision and more to do with the clinician’s broader sense of their own role, their relationship with the patient, and the institutional and professional context within which that relationship takes place.

Second, participants’ own discussions of defensive practice tend to emphasise concerns with documentation and professional autonomy as much as, or more than, identifiable changes to treatment. The threat of litigation and complaints opens onto a broader set of concerns about institutional or social pressures on clinicians—particularly involving regulation and deskilling—which are seen as part of defensive practice, but which often have only a very tenuous relationship with litigation risk. Clinicians are aware of the stock examples of defensive overtreatment and overdiagnosis, but their practical concerns seem often to have more to do with overdocumentation and patient relationships.

These findings, then, call for more nuance around the narrative in which defensive practice is a key causal pathway linking litigation risk to inappropriate treatment and excess costs. On the one hand, decisions which clinicians define as defensive practice may not be directly related to the objective probability of litigation or complaints; rather, these risks become salient to practice only as mediated through a broader set of concerns and narratives. On the other, if the lived experience of defensive practice (at least outside of obstetrics) often relates more to administrative processes such as documentation than to specific treatment decisions, then the very high rates of self-reported defensive practice found in survey studies may not translate into suboptimal care at a large scale. (This said, concern about impacts on care and costs may also relate to overdiagnosis, which was more often mentioned than overtreatment in the studies.)

This conclusion is in line with at least some strands of the policy literature, which have emphasised that measures focused narrowly on litigation risk, such as tort reform (eg, compensation caps), may have limited impact if the broader cultural and regulatory contexts of defensive practice are not addressed.^43^,^45^ The quantitative research literature is also less than conclusive as to whether litigation risk is actually linked to care or cost outcomes. Agarwal *et al*’s systematic review of tort reform measures found that they were associated with reductions in healthcare expenditure in about half of the studies, with half finding no significant effect.^46^ Cross-sectional studies linking malpractice liability costs to healthcare expenditure have generally found little or no significant correlation,^47 48^ although studies using more specific outcomes, such as rates of caesarean section, have sometimes found a relationship.^49 50^ Even where a correlation is observed, it cannot automatically be concluded that defensive practice is the key causal link, given that the outcomes measured represent a snapshot of a highly complex system, and other differences (eg, in healthcare provision or characteristics of the patient population) could play an important role.

Hence, the qualitative evidence, which suggests that the links between litigation risk and care outcomes are more complex in practice than initially apparent, may not be wholly misaligned with the quantitative literature. However, a broader perspective on the findings of this review seems to be called for. Such a perspective might be less inclined to focus on the negative aspects of changes such as improved transparency in clinical governance, guideline-based care or a greater willingness in the broader society to question the self-perception of elite professional groups. The review findings suggest not only that the enacted reality of defensive practice may substantially diverge from the theoretical narrative, but also that it may call for more nuanced critical engagement.

## supplementary material

10.1136/bmjopen-2024-085673 online supplemental file 1

10.1136/bmjopen-2024-085673 online supplemental file 2

10.1136/bmjopen-2024-085673 online supplemental file 3

## Data Availability

Data are available on reasonable request.
