# Supplementary material for: Defensive healthcare practice: systematic review of qualitative evidence
Source: BMJ Open. 2024 Jul 18;14(7):e085673. doi: 10.1136/bmjopen-2024-085673 (PMC11261683; doi:10.1136/bmjopen-2024-085673)
Supplement: online supplemental file 1 [file bmjopen-14-7-s001.pdf]

Defensive healthcare practice: Systematic review of qualitative evidence

Appendix A. Full search strategies

MEDLINE(R) ALL
via Ovid http://ovidsp.ovid.com/
1946 to January 06, 2020
Searched on: 7th January 2020
Records retrieved: 3650

1 Defensive Medicine/ (1228)
2 (defensive$ adj4 (medicine or medical)).ti,ab,kf. (549)
3 (defensive$ adj4 practic$).ti,ab,kf. (272)
4 (defensive$ adj4 decision$).ti,ab,kf. (15)
5 (defensive$ adj4 work$).ti,ab,kf. (67)
6 or/1-5 (1651)
7 Liability, Legal/ (15518)
8 Jurisprudence/ (29681)
9 Malpractice/ (27599)
10 Professional Misconduct/ (3305)
11 Employee Discipline/ (1483)
12 "Compensation and Redress"/ (2958)
13 or/7-12 (69771)
14 Fear/ (30785)
15 13 and 14 (143)
16 (fear$ adj4 (legal$ or liabilit$ or complaint$ or litigat$ or claim$ or lawsuit$ or prosecut$ or compensation$ or damages or "being sued" or malpractice or negligen$ or misconduct)).ti,ab. (975)
17 (anxiet$ adj4 (legal$ or liabilit$ or complaint$ or litigat$ or claim$ or lawsuit$ or prosecut$ or compensation$ or damages or "being sued" or malpractice or negligen$ or misconduct)).ti,ab. (828)
18 ((worry or worrie$) adj4 (legal$ or liabilit$ or complaint$ or litigat$ or claim$ or lawsuit$ or prosecut$ or compensation$ or damages or "being sued" or malpractice or negligen$ or misconduct)).ti,ab. (120)
19 (apprehensi$ adj4 (legal$ or liabilit$ or complaint$ or litigat$ or claim$ or lawsuit$ or prosecut$ or compensation$ or damages or "being sued" or malpractice or negligen$ or misconduct)).ti,ab. (26)
20 (afraid adj4 (legal$ or liabilit$ or complaint$ or litigat$ or claim$ or lawsuit$ or prosecut$ or compensation$ or damages or "being sued" or malpractice or negligen$ or misconduct)).ti,ab. (11)
21 (dread$ adj4 (legal$ or liabilit$ or complaint$ or litigat$ or claim$ or lawsuit$ or prosecut$ or compensation$ or damages or "being sued" or malpractice or negligen$ or misconduct)).ti,ab. (9)
22 (threat$ adj4 (legal$ or liabilit$ or complaint$ or litigat$ or claim$ or lawsuit$ or prosecut$ or compensation$ or damages or "being sued" or malpractice or negligen$ or misconduct)).ti,ab. (639)
23 (expos$ adj4 (legal$ or liabilit$ or complaint$ or litigat$ or claim$ or lawsuit$ or prosecut$ or compensation$ or damages or "being sued" or malpractice or negligen$ or misconduct)).ti,ab. (1697)
24 (avoid$ adj4 (legal$ or liabilit$ or complaint$ or litigat$ or claim$ or lawsuit$ or prosecut$ or compensation$ or damages or "being sued" or malpractice or negligen$ or misconduct)).ti,ab. (1610)
25 ((fear$ or anxiet$ or worry or worrie$ or apprehensi$ or afraid or dread$ or threat$ or expos$ or avoid$) adj4 disciplin$ adj4 (action$ or measure or measures or procedure$ or proceeding$ or process$ or sanction$)).ti,ab. (36)

26 ((fear$ or anxiet$ or worry or worrie$ or apprehensi$ or afraid or dread$ or threat$ or expos$ or avoid$) adj4 (professional$ or formal$ or external$ or official$) adj4 regulat$).ti,ab. (29)
27 or/16-26 (5818)
28 15 or 27 (5908)
29 Professional Practice/ (16569)
30 Practice Patterns, Physicians'/ (57658)
31 Practice Patterns, Dentists'/ (2304)
32 Institutional Practice/ (1236)
33 Professional Autonomy/ (9418)
34 or/29-33 (86199)
35 13 and 34 (1544)
36 (practice$ adj6 (legal$ or liabilit$ or complaint$ or litigat$ or claim$ or lawsuit$ or prosecut$ or compensation$ or damages or "being sued" or malpractice or negligen$ or misconduct)).ti,ab. (5184)
37 (behav$ adj6 (legal$ or liabilit$ or complaint$ or litigat$ or claim$ or lawsuit$ or prosecut$ or compensation$ or damages or "being sued" or malpractice or negligen$ or misconduct)).ti,ab. (2945)
38 (autonom$ adj6 (legal$ or liabilit$ or complaint$ or litigat$ or claim$ or lawsuit$ or prosecut$ or compensation$ or damages or "being sued" or malpractice or negligen$ or misconduct)).ti,ab. (574)
39 ((professional$ or formal$ or external$ or official$) adj6 regulat$ adj6 (practice$ or behav$ or autonom$)).ti,ab. (436)
40 (disciplinary adj6 (action$ or measure or measures or procedure$ or proceeding$ or process$ or sanction$) adj6 (practice$ or behav$ or autonom$)).ti,ab. (76)
41 or/36-40 (9120)
42 35 or 41 (10492)
43 (defensive$ adj3 (act or acts or action$ or approach$ or strateg$)).ti,ab. (834)
44 (legal$ or liabilit$ or complaint$ or litigat$ or claim$ or lawsuit$ or prosecut$ or compensation$ compensation$ or damages or "being sued" or malpractice or negligen$ or misconduct or regulat$ or disciplin$).ti,ab. (2140997)
45 13 or 44 (2183723)
46 43 and 45 (124)
47 6 or 28 or 42 or 46 (17382)
48 exp Qualitative Research/ (51111)
49 Interview/ (28868)
50 Focus Groups/ (28404)
51 Qualitative.mp. (233907)
52 Interview$.mp. (375647)
53 Experience$.mp. (1032821)
54 Focus group$.ti,ab. (43896)
55 (attitude$ or belief$ or believ$ or opinion$ or perceiv$ or perception$ or preference$ or view or views or viewpoint$).ti,ab. (1282551)
56 or/48-55 (2496195)
57 47 and 56 (4742)
58 (mixed method$ or multimethod$ or multi-method$ or multi method$).mp. (23059)
59 realist synthes$.ti,ab. (198)
60 (meta-synthes$ or metasynthes$).ti,ab. (1131)
61 (meta-ethnograph$ or metaethnograph$).ti,ab. (536)
62 (meta-study or metastudy).ti,ab. (99)
63 realist review$.ti,ab. (305)
64 or/58-63 (24978)
65 47 and 64 (65)
66 57 or 65 (4750)

67 exp animals/ not humans/ (4660757)
68 66 not 67 (4655)
69 limit 68 to yr="2000 -Current" (3650)

Embase
via Ovid http://ovidsp.ovid.com/
1974 to 2020 January 03
Searched on: 7th January 2020
Records retrieved: 6378

1 defensive medicine/ (382)
2 (defensive$ adj4 (medicine or medical)).ti,ab,kw. (663)
3 (defensive$ adj4 practice$).ti,ab,kw. (322)
4 (defensive$ adj4 decision$).ti,ab,kw. (17)
5 (defensive$ adj4 work$).ti,ab,kw. (75)
6 1 or 2 or 3 or 4 or 5 (1046)
7 legal liability/ (16441)
8 medical liability/ (4388)
9 jurisprudence/ (24173)
10 malpractice/ (31981)
11 professional misconduct/ (3808)
12 negligence/ (4317)
13 law suit/ (11377)
14 compensation/ (14152)
15 medicolegal aspect/ (26000)
16 7 or 8 or 9 or 10 or 11 or 12 or 13 or 14 or 15 (114511)
17 fear/ (58964)
18 16 and 17 (615)
19 (fear$ adj4 (legal$ or liabilit$ or complaint$ or litigat$ or claim$ or lawsuit$ or prosecut$ or compensation$ or damages or "being sued" or malpractice or negligen$ or misconduct)).ti,ab. (1232)
20 (anxiet$ adj4 (legal$ or liabilit$ or complaint$ or litigat$ or claim$ or lawsuit$ or prosecut$ or compensation$ or damages or "being sued" or malpractice or negligen$ or misconduct)).ti,ab. (1242)
21 ((worry or worrie$) adj4 (legal$ or liabilit$ or complaint$ or litigat$ or claim$ or lawsuit$ or prosecut$ or compensation$ or damages or "being sued" or malpractice or negligen$ or misconduct)).ti,ab. (174)
22 (apprehensi$ adj4 (legal$ or liabilit$ or complaint$ or litigat$ or claim$ or lawsuit$ or prosecut$ or compensation$ or damages or "being sued" or malpractice or negligen$ or misconduct)).ti,ab. (29)
23 (afraid adj4 (legal$ or liabilit$ or complaint$ or litigat$ or claim$ or lawsuit$ or prosecut$ or compensation$ or damages or "being sued" or malpractice or negligen$ or misconduct)).ti,ab. (16)
24 (dread$ adj4 (legal$ or liabilit$ or complaint$ or litigat$ or claim$ or lawsuit$ or prosecut$ or compensation$ or damages or "being sued" or malpractice or negligen$ or misconduct)).ti,ab. (11)
25 (threat$ adj4 (legal$ or liabilit$ or complaint$ or litigat$ or claim$ or lawsuit$ or prosecut$ or compensation$ or damages or "being sued" or malpractice or negligen$ or misconduct)).ti,ab. (743)
26 (expos$ adj4 (legal$ or liabilit$ or complaint$ or litigat$ or claim$ or lawsuit$ or prosecut$ or compensation$ or damages or "being sued" or malpractice or negligen$ or misconduct)).ti,ab. (2147)
27 (avoid$ adj4 (legal$ or liabilit$ or complaint$ or litigat$ or claim$ or lawsuit$ or prosecut$ or compensation$ or damages or "being sued" or malpractice or negligen$ or misconduct)).ti,ab. (2093)
28 ((fear$ or anxiet$ or worry or worrie$ or apprehensi$ or afraid or dread$ or threat$ or expos$ or avoid$) adj4 disciplin$ adj4 (action$ or measure or measures or procedure$ or proceeding$ or process$ or sanction$)).ti,ab. (42)
29 ((fear$ or anxiet$ or worry or worrie$ or apprehensi$ or afraid or dread$ or threat$ or expos$ or avoid$) adj4 (professional$ or formal$ or external$ or official$) adj4 regulat$).ti,ab. (35)
30 19 or 20 or 21 or 22 or 23 or 24 or 25 or 26 or 27 or 28 or 29 (7575)
31 18 or 30 (7963)

32 professional practice/ (58627)
33 clinical practice/ (275280)
34 medical practice/ (86907)
35 32 or 33 or 34 (411113)
36 16 and 35 (9503)
37 (practice$ adj6 (legal$ or liabilit$ or complaint$ or litigat$ or claim$ or lawsuit$ or prosecut$ or compensation$ or damages or "being sued" or malpractice or negligen$ or misconduct)).ti,ab. (6568)
38 (behav$ adj6 (legal$ or liabilit$ or complaint$ or litigat$ or claim$ or lawsuit$ or prosecut$ or compensation$ or damages or "being sued" or malpractice or negligen$ or misconduct)).ti,ab. (3833)
39 (autonom$ adj6 (legal$ or liabilit$ or complaint$ or litigat$ or claim$ or lawsuit$ or prosecut$ or compensation$ or damages or "being sued" or malpractice or negligen$ or misconduct)).ti,ab. (750)
40 ((professional$ or formal$ or external$ or official$) adj6 regulat$ adj6 (practice$ or behav$ or autonom$)).ti,ab. (521)
41 (disciplinary adj6 (action$ or measure or measures or procedure$ or proceeding$ or process$ or sanction$) adj6 (practice$ or behav$ or autonom$)).ti,ab. (98)
42 37 or 38 or 39 or 40 or 41 (11648)
43 36 or 42 (20509)
44 (defensive$ adj3 (act or acts or action$ or approach$ or strateg$)).ti,ab. (941)
45 (legal$ or liabilit$ or complaint$ or litigat$ or claim$ or lawsuit$ or prosecut$ or compensation$ compensation$ or damages or "being sued" or malpractice or negligen$ or misconduct or regulat$ or disciplin$).ti,ab. (2689239)
46 45 or 16 (2762527)
47 44 and 46 (158)
48 6 or 31 or 43 or 47 (28613)
49 exp qualitative research/ (70735)
50 interview$.mp. or interview/ or semi structured interview/ or structured interview/ or exp telephone interview/ or unstructured interview/ (485827)
51 (focus adj group$).mp. (55214)
52 qualitative.mp. (307073)
53 Experience$.mp. (1456213)
54 (attitude$ or belief$ or believ$ or opinion$ or perceiv$ or perception$ or preference$ or view or views or viewpoint$).ti,ab. (1595190)
55 49 or 50 or 51 or 52 or 53 or 54 (3280244)
56 48 and 55 (7705)
57 (mixed method$ or multimethod$ or multi-method$ or multi method$).mp. (27517)
58 realist synthes$.ti,ab. (187)
59 (meta-synthes$ or metasynthes$).ti,ab. (1238)
60 (meta-ethnograph$ or metaethnograph$).ti,ab. (587)
61 (meta-study or metastudy).ti,ab. (113)
62 realist review$.ti,ab. (324)
63 57 or 58 or 59 or 60 or 61 or 62 (29588)
64 48 and 63 (88)
65 56 or 64 (7719)
66 (animal/ or animal experiment/ or animal model/ or animal tissue/ or nonhuman/) not exp human/ (5911147)
67 65 not 66 (7576)
68 limit 67 to yr="2000 -Current" (6378)

PsycINFO
via Ovid http://ovidsp.ovid.com/

1987 to December Week 5, 2019
Searched on: 7th January 2020
Records retrieved: 3604

1 (defensive$ adj4 (medicine or medical)).ti,ab,id. (63)
2 (defensive$ adj4 practic$).ti,ab,id. (124)
3 (defensive$ adj4 decision$).ti,ab,id. (28)
4 (defensive$ adj4 work$).ti,ab,id. (122)
5 1 or 2 or 3 or 4 (298)
6 professional liability/ (1845)
7 litigation/ (1319)
8 legal processes/ (12598)
9 6 or 7 or 8 (14964)
10 fear/ (14896)
11 9 and 10 (28)
12 (fear$ adj4 (legal$ or liabilit$ or complaint$ or litigat$ or claim$ or lawsuit$ or prosecut$ or compensation$ or damages or "being sued" or malpractice or negligen$ or misconduct)).ti,ab. (388)
13 (anxiet$ adj4 (legal$ or liabilit$ or complaint$ or litigat$ or claim$ or lawsuit$ or prosecut$ or compensation$ or damages or "being sued" or malpractice or negligen$ or misconduct)).ti,ab. (851)
14 ((worry or worrie$) adj4 (legal$ or liabilit$ or complaint$ or litigat$ or claim$ or lawsuit$ or prosecut$ or compensation$ or damages or "being sued" or malpractice or negligen$ or misconduct)).ti,ab. (72)
15 (apprehensi$ adj4 (legal$ or liabilit$ or complaint$ or litigat$ or claim$ or lawsuit$ or prosecut$ or compensation$ or damages or "being sued" or malpractice or negligen$ or misconduct)).ti,ab. (19)
16 (afraid adj4 (legal$ or liabilit$ or complaint$ or litigat$ or claim$ or lawsuit$ or prosecut$ or compensation$ or damages or "being sued" or malpractice or negligen$ or misconduct)).ti,ab. (5)
17 (dread$ adj4 (legal$ or liabilit$ or complaint$ or litigat$ or claim$ or lawsuit$ or prosecut$ or compensation$ or damages or "being sued" or malpractice or negligen$ or misconduct)).ti,ab. (1)
18 (threat$ adj4 (legal$ or liabilit$ or complaint$ or litigat$ or claim$ or lawsuit$ or prosecut$ or compensation$ or damages or "being sued" or malpractice or negligen$ or misconduct)).ti,ab. (342)
19 (expos$ adj4 (legal$ or liabilit$ or complaint$ or litigat$ or claim$ or lawsuit$ or prosecut$ or compensation$ or damages or "being sued" or malpractice or negligen$ or misconduct)).ti,ab. (491)
20 (avoid$ adj4 (legal$ or liabilit$ or complaint$ or litigat$ or claim$ or lawsuit$ or prosecut$ or compensation$ or damages or "being sued" or malpractice or negligen$ or misconduct)).ti,ab. (582)
21 ((fear$ or anxiet$ or worry or worrie$ or apprehensi$ or afraid or dread$ or threat$ or expos$ or avoid$) adj4 disciplin$ adj4 (action$ or measure or measures or procedure$ or proceeding$ or process$ or sanction$)).ti,ab. (22)
22 ((fear$ or anxiet$ or worry or worrie$ or apprehensi$ or afraid or dread$ or threat$ or expos$ or avoid$) adj4 (professional$ or formal$ or external$ or official$) adj4 regulat$).ti,ab. (13)
23 12 or 13 or 14 or 15 or 16 or 17 or 18 or 19 or 20 or 21 or 22 (2721)
24 11 or 23 (2737)
25 clinical practice/ (18945)
26 professional role/ (339)
27 autonomy/ (6583)
28 25 or 26 or 27 (25824)
29 9 and 28 (202)
30 (practice$ adj6 (legal$ or liabilit$ or complaint$ or litigat$ or claim$ or lawsuit$ or prosecut$ or compensation$ or damages or "being sued" or malpractice or negligen$ or misconduct)).ti,ab. (3225)
31 (behav$ adj6 (legal$ or liabilit$ or complaint$ or litigat$ or claim$ or lawsuit$ or prosecut$ or compensation$ or damages or "being sued" or malpractice or negligen$ or misconduct)).ti,ab. (3549)

32 (autonom$ adj6 (legal$ or liabilit$ or complaint$ or litigat$ or claim$ or lawsuit$ or prosecut$ or compensation$ or damages or "being sued" or malpractice or negligen$ or misconduct)).ti,ab. (341)
33 ((professional$ or formal$ or external$ or official$) adj6 regulat$ adj6 (practice$ or behav$ or autonom$)).ti,ab. (405)
34 (disciplinary adj6 (action$ or measure or measures or procedure$ or proceeding$ or process$ or sanction$) adj6 (practice$ or behav$ or autonom$)).ti,ab. (111)
35 30 or 31 or 32 or 33 or 34 (7514)
36 29 or 35 (7647)
37 (defensive$ adj3 (act or acts or action$ or approach$ or strateg$)).ti,ab. (718)
38 (legal$ or liabilit$ or complaint$ or litigat$ or claim$ or lawsuit$ or prosecut$ or compensation$ compensation$ or damages or "being sued" or malpractice or negligen$ or misconduct or regulat$ or disciplin$).ti,ab. (335793)
39 9 or 38 (339367)
40 37 and 39 (101)
41 5 or 24 or 36 or 40 (10496)
42 exp qualitative methods/ (14143)
43 qualitative measures/ (45)
44 interviews/ or semi-structured interview/ or exp interviewing/ (9906)
45 qualitative.tw. (155055)
46 interview$.tw. (292619)
47 experience$.tw. (551722)
48 focus group$.ti,ab. (33808)
49 (attitude$ or belief$ or believ$ or opinion$ or perceiv$ or perception$ or preference$ or view or views or viewpoint$).ti,ab. (832208)
50 42 or 43 or 44 or 45 or 46 or 47 or 48 or 49 (1382427)
51 41 and 50 (4360)
52 (mixed method$ or multimethod$ or multi-method$ or multi method$).mp. (27969)
53 realist synthes$.ti,ab. (76)
54 (meta-synthes$ or metasynthes$).ti,ab. (749)
55 (meta-ethnograph$ or metaethnograph$).ti,ab. (305)
56 (meta-study or metastudy).ti,ab. (83)
57 realist review$.ti,ab. (69)
58 52 or 53 or 54 or 55 or 56 or 57 (29064)
59 41 and 58 (85)
60 51 or 59 (4369)
61 (rat or rats or mouse or mice or hamster or hamsters or animal or animals or dog or dogs or cat or cats or bovine or sheep).ti,ab,sh. (260599)
62 60 not 61 (4285)
63 limit 62 to yr="2000 -Current" (3604)

Allied and Complementary Medicine (AMED)
via Ovid http://ovidsp.ovid.com/
1985 to December 2019
Searched on: 7th January 2020
Records retrieved: 422

1 (defensive$ adj4 (medicine or medical)).mp. (2)
2 (defensive$ adj4 practic$).mp. (1)
3 (defensive$ adj4 decision$).mp. (3)
4 (defensive$ adj4 work$).mp. (2)

5 1 or 2 or 3 or 4 (8)
6 jurisprudence/ (1155)
7 malpractice/ (134)
8 6 or 7 (1264)
9 fear/ (514)
10 8 and 9 (0)
11 (fear$ adj4 (legal$ or liabilit$ or complaint$ or litigat$ or claim$ or lawsuit$ or prosecut$ or compensation$ or damages or "being sued" or malpractice or negligen$ or misconduct)).mp. (22)
12 (anxiet$ adj4 (legal$ or liabilit$ or complaint$ or litigat$ or claim$ or lawsuit$ or prosecut$ or compensation$ or damages or "being sued" or malpractice or negligen$ or misconduct)).mp. (25)
13 ((worry or worrie$) adj4 (legal$ or liabilit$ or complaint$ or litigat$ or claim$ or lawsuit$ or prosecut$ or compensation$ or damages or "being sued" or malpractice or negligen$ or misconduct)).mp. (0)
14 (apprehensi$ adj4 (legal$ or liabilit$ or complaint$ or litigat$ or claim$ or lawsuit$ or prosecut$ or compensation$ or damages or "being sued" or malpractice or negligen$ or misconduct)).mp. (1)
15 (afraid adj4 (legal$ or liabilit$ or complaint$ or litigat$ or claim$ or lawsuit$ or prosecut$ or compensation$ or damages or "being sued" or malpractice or negligen$ or misconduct)).mp. (0)
16 (dread$ adj4 (legal$ or liabilit$ or complaint$ or litigat$ or claim$ or lawsuit$ or prosecut$ or compensation$ or damages or "being sued" or malpractice or negligen$ or misconduct)).mp. (0)
17 (threat$ adj4 (legal$ or liabilit$ or complaint$ or litigat$ or claim$ or lawsuit$ or prosecut$ or compensation$ or damages or "being sued" or malpractice or negligen$ or misconduct)).mp. (10)
18 (expos$ adj4 (legal$ or liabilit$ or complaint$ or litigat$ or claim$ or lawsuit$ or prosecut$ or compensation$ or damages or "being sued" or malpractice or negligen$ or misconduct)).mp. (24)
19 (avoid$ adj4 (legal$ or liabilit$ or complaint$ or litigat$ or claim$ or lawsuit$ or prosecut$ or compensation$ or damages or "being sued" or malpractice or negligen$ or misconduct)).mp. (38)
20 ((fear$ or anxiet$ or worry or worrie$ or apprehensi$ or afraid or dread$ or threat$ or expos$ or avoid$) adj4 disciplin$ adj4 (action$ or measure or measures or procedure$ or proceeding$ or process$ or sanction$)).mp. (0)
21 ((fear$ or anxiet$ or worry or worrie$ or apprehensi$ or afraid or dread$ or threat$ or expos$ or avoid$) adj4 (professional$ or formal$ or external$ or official$) adj4 regulat$).mp. (0)
22 11 or 12 or 13 or 14 or 15 or 16 or 17 or 18 or 19 or 20 or 21 (117)
23 10 or 22 (117)
24 professional practice/ (7684)
25 8 and 24 (99)
26 (practice$ adj6 (legal$ or liabilit$ or complaint$ or litigat$ or claim$ or lawsuit$ or prosecut$ or compensation$ or damages or "being sued" or malpractice or negligen$ or misconduct)).mp. (273)
27 (behav$ adj6 (legal$ or liabilit$ or complaint$ or litigat$ or claim$ or lawsuit$ or prosecut$ or compensation$ or damages or "being sued" or malpractice or negligen$ or misconduct)).mp. (77)
28 (autonom$ adj6 (legal$ or liabilit$ or complaint$ or litigat$ or claim$ or lawsuit$ or prosecut$ or compensation$ or damages or "being sued" or malpractice or negligen$ or misconduct)).mp. (29)
29 ((professional$ or formal$ or external$ or official$) adj6 regulat$ adj6 (practice$ or behav$ or autonom$)).mp. (19)
30 (disciplinary adj6 (action$ or measure or measures or procedure$ or proceeding$ or process$ or sanction$) adj6 (practice$ or behav$ or autonom$)).mp. (7)
31 26 or 27 or 28 or 29 or 30 (394)
32 25 or 31 (481)
33 (defensive$ adj3 (act or acts or action$ or approach$ or strateg$)).mp. (15)
34 (legal$ or liabilit$ or complaint$ or litigat$ or claim$ or lawsuit$ or prosecut$ or compensation$ compensation$ or damages or "being sued" or malpractice or negligen$ or misconduct or regulat$ or disciplin$).mp. (12133)
35 8 or 34 (12949)

36 33 and 35 (3)
37 5 or 23 or 32 or 36 (596)
38 limit 37 to yr="2000 -Current" (422)

Maternity and Infant Care
via Ovid http://ovidsp.ovid.com/
1971 to November 2019
Searched on: 7th January 2020
Records retrieved: 187

1 (defensive$ adj4 (medicine or medical)).mp. (31)
2 (defensive$ adj4 practic$).mp. (32)
3 (defensive$ adj4 decision$).mp. (2)
4 (defensive$ adj4 work$).mp. (1)
5 1 or 2 or 3 or 4 (56)
6 (fear$ adj4 (legal$ or liabilit$ or complaint$ or litigat$ or claim$ or lawsuit$ or prosecut$ or compensation$ or damages or "being sued" or malpractice or negligen$ or misconduct)).mp. (112)
7 (anxiet$ adj4 (legal$ or liabilit$ or complaint$ or litigat$ or claim$ or lawsuit$ or prosecut$ or compensation$ or damages or "being sued" or malpractice or negligen$ or misconduct)).mp. (9)
8 ((worry or worrie$) adj4 (legal$ or liabilit$ or complaint$ or litigat$ or claim$ or lawsuit$ or prosecut$ or compensation$ or damages or "being sued" or malpractice or negligen$ or misconduct)).mp. (4)
9 (apprehensi$ adj4 (legal$ or liabilit$ or complaint$ or litigat$ or claim$ or lawsuit$ or prosecut$ or compensation$ or damages or "being sued" or malpractice or negligen$ or misconduct)).mp. (0)
10 (afraid adj4 (legal$ or liabilit$ or complaint$ or litigat$ or claim$ or lawsuit$ or prosecut$ or compensation$ or damages or "being sued" or malpractice or negligen$ or misconduct)).mp. (3)
11 (dread$ adj4 (legal$ or liabilit$ or complaint$ or litigat$ or claim$ or lawsuit$ or prosecut$ or compensation$ or damages or "being sued" or malpractice or negligen$ or misconduct)).mp. (1)
12 (threat$ adj4 (legal$ or liabilit$ or complaint$ or litigat$ or claim$ or lawsuit$ or prosecut$ or compensation$ or damages or "being sued" or malpractice or negligen$ or misconduct)).mp. (35)
13 (expos$ adj4 (legal$ or liabilit$ or complaint$ or litigat$ or claim$ or lawsuit$ or prosecut$ or compensation$ or damages or "being sued" or malpractice or negligen$ or misconduct)).mp. (36)
14 (avoid$ adj4 (legal$ or liabilit$ or complaint$ or litigat$ or claim$ or lawsuit$ or prosecut$ or compensation$ or damages or "being sued" or malpractice or negligen$ or misconduct)).mp. (59)
15 ((fear$ or anxiet$ or worry or worrie$ or apprehensi$ or afraid or dread$ or threat$ or expos$ or avoid$) adj4 disciplin$ adj4 (action$ or measure or measures or procedure$ or proceeding$ or process$ or sanction$)).mp. (5)
16 ((fear$ or anxiet$ or worry or worrie$ or apprehensi$ or afraid or dread$ or threat$ or expos$ or avoid$) adj4 (professional$ or formal$ or external$ or official$) adj4 regulat$).mp. (0)
17 6 or 7 or 8 or 9 or 10 or 11 or 12 or 13 or 14 or 15 or 16 (256)
18 (practice$ adj6 (legal$ or liabilit$ or complaint$ or litigat$ or claim$ or lawsuit$ or prosecut$ or compensation$ or damages or "being sued" or malpractice or negligen$ or misconduct)).mp. (301)
19 (behav$ adj6 (legal$ or liabilit$ or complaint$ or litigat$ or claim$ or lawsuit$ or prosecut$ or compensation$ or damages or "being sued" or malpractice or negligen$ or misconduct)).mp. (41)
20 (autonom$ adj6 (legal$ or liabilit$ or complaint$ or litigat$ or claim$ or lawsuit$ or prosecut$ or compensation$ or damages or "being sued" or malpractice or negligen$ or misconduct)).mp. (25)
21 ((professional$ or formal$ or external$ or official$) adj6 regulat$ adj6 (practice$ or behav$ or autonom$)).mp. (75)
22 (disciplinary adj6 (action$ or measure or measures or procedure$ or proceeding$ or process$ or sanction$) adj6 (practice$ or behav$ or autonom$)).mp. (5)
23 18 or 19 or 20 or 21 or 22 (441)

24 (defensive$ adj3 (act or acts or action$ or approach$ or strateg$)).mp. (2)
25 (legal$ or liabilit$ or complaint$ or litigat$ or claim$ or lawsuit$ or prosecut$ or compensation$ compensation$ or damages or "being sued" or malpractice or negligen$ or misconduct or regulat$ or disciplin$).mp. (14449)
26 24 and 25 (0)
27 5 or 17 or 23 or 26 (691)
28 qualitative.mp. (6787)
29 interview$.mp. (13986)
30 experience$.mp. (31631)
31 focus group$.mp. (2322)
32 (attitude$ or belief$ or believ$ or opinion$ or perceiv$ or perception$ or preference$ or view or views or viewpoint$).ti,ab. (26296)
33 28 or 29 or 30 or 31 or 32 (58304)
34 27 and 33 (237)
35 (mixed method$ or multimethod$ or multi-method$ or multi method$).mp. (921)
36 realist synthes$.ti,ab. (6)
37 (meta-synthes$ or metasynthes$).ti,ab. (112)
38 (meta-ethnograph$ or metaethnograph$).ti,ab. (75)
39 (meta-study or metastudy).ti,ab. (5)
40 realist review$.ti,ab. (12)
41 35 or 36 or 37 or 38 or 39 or 40 (1088)
42 27 and 41 (5)
43 34 or 42 (237)
44 limit 43 to yr="2000 -Current" (187)

Cumulative Index to Nursing & Allied Health (CINAHL Complete)
via Ebsco https://www.ebscohost.com/
Inception to 7th January 2020
Searched on: 7th January 2020
Records retrieved: 2559

S1	TI (defensive* N4 (medicine or medical)) OR AB (defensive* N4 (medicine or medical))	196
S2	TI defensive* N4 practic* OR AB defensive* N4 practic*	149
S3	TI defensive* N4 decision* OR AB defensive* N4 decision*	10
S4	TI defensive* N4 work* OR AB defensive* N4 work*	34
S5	S1 OR S2 OR S3 OR S4	308
S6	(MH "Liability, Legal")	13,753
S7	(MH "Jurisprudence")	7,206
S8	(MH "Malpractice")	9,251
S9	(MH "Professional Misconduct")	4,485
S10	(MH "Employee Discipline")	1,262
S11	(MH "Damages, Legal")	2,557
S12	(MH "Professional Regulation")	6,072
S13	(MH "Legal Procedure")	5,422
S14	(MH "Negligence")	4,298
S15	(S6 OR S7 OR S8 OR S9 OR S10 OR S11 OR S12 OR S13 OR S14)	44,394
S16	(MH "Fear")	11,679
S17	S15 AND S16	120
S18	TI (fear* N4 (legal* or liabilit* or complaint* or litigat* or claim* or lawsuit* or prosecut* or compensation* or damages or "being sued" or malpractice or negligen* or misconduct)) OR AB (fear* N4 (legal* or liabilit* or complaint* or litigat* or claim* or lawsuit* or prosecut* or compensation* or damages or "being sued" or malpractice or negligen* or misconduct))	544
S19	TI (anxiet* N4 (legal* or liabilit* or complaint* or litigat* or claim* or lawsuit* or prosecut* or compensation* or damages or "being sued" or malpractice or negligen* or misconduct)) OR AB (	

anxiet* N4 (legal* or liabilit* or complaint* or litigat* or claim* or lawsuit* or prosecut* or compensation* or damages or "being sued" or malpractice or negligen* or misconduct)) 357

S20 TI ((worry or worrie*) N4 (legal* or liabilit* or complaint* or litigat* or claim* or lawsuit* or prosecut* or compensation* or damages or "being sued" or malpractice or negligen* or misconduct)) OR AB ((worry or worrie*) N4 (legal* or liabilit* or complaint* or litigat* or claim* or lawsuit* or prosecut* or compensation* or damages or "being sued" or malpractice or negligen* or misconduct)) 82

S21 TI (apprehensi* N4 (legal* or liabilit* or complaint* or litigat* or claim* or lawsuit* or prosecut* or compensation* or damages or "being sued" or malpractice or negligen* or misconduct)) OR AB (apprehensi* N4 (legal* or liabilit* or complaint* or litigat* or claim* or lawsuit* or prosecut* or compensation* or damages or "being sued" or malpractice or negligen* or misconduct)) 10

S22 TI (afraid N4 (legal* or liabilit* or complaint* or litigat* or claim* or lawsuit* or prosecut* or compensation* or damages or "being sued" or malpractice or negligen* or misconduct)) OR AB (afraid N4 (legal* or liabilit* or complaint* or litigat* or claim* or lawsuit* or prosecut* or compensation* or damages or "being sued" or malpractice or negligen* or misconduct)) 13

S23 TI (dread* N4 (legal* or liabilit* or complaint* or litigat* or claim* or lawsuit* or prosecut* or compensation* or damages or "being sued" or malpractice or negligen* or misconduct)) OR AB (dread* N4 (legal* or liabilit* or complaint* or litigat* or claim* or lawsuit* or prosecut* or compensation* or damages or "being sued" or malpractice or negligen* or misconduct)) 3

S24 TI (threat* N4 (legal* or liabilit* or complaint* or litigat* or claim* or lawsuit* or prosecut* or compensation* or damages or "being sued" or malpractice or negligen* or misconduct)) OR AB (threat* N4 (legal* or liabilit* or complaint* or litigat* or claim* or lawsuit* or prosecut* or compensation* or damages or "being sued" or malpractice or negligen* or misconduct)) 421

S25 TI (expos* N4 (legal* or liabilit* or complaint* or litigat* or claim* or lawsuit* or prosecut* or compensation* or damages or "being sued" or malpractice or negligen* or misconduct)) OR AB (expos* N4 (legal* or liabilit* or complaint* or litigat* or claim* or lawsuit* or prosecut* or compensation* or damages or "being sued" or malpractice or negligen* or misconduct)) 1,087

S26 TI (avoid* N4 (legal* or liabilit* or complaint* or litigat* or claim* or lawsuit* or prosecut* or compensation* or damages or "being sued" or malpractice or negligen* or misconduct)) OR AB (avoid* N4 (legal* or liabilit* or complaint* or litigat* or claim* or lawsuit* or prosecut* or compensation* or damages or "being sued" or malpractice or negligen* or misconduct)) 1,362

S27 TI ((fear* or anxiet* or worry or worrie* or apprehensi* or afraid or dread* or threat* or expos* or avoid*) N4 disciplin* N4 (action* or measure or measures or procedure* or proceeding* or process* or sanction*)) OR AB ((fear* or anxiet* or worry or worrie* or apprehensi* or afraid or dread* or threat* or expos* or avoid*) N4 disciplin* N4 (action* or measure or measures or procedure* or proceeding* or process* or sanction*)) 35

S28 TI ((fear* or anxiet* or worry or worrie* or apprehensi* or afraid or dread* or threat* or expos* or avoid*) N4 (professional* or formal* or external* or official*) N4 regulat*)) OR AB ((fear* or anxiet* or worry or worrie* or apprehensi* or afraid or dread* or threat* or expos* or avoid*) N4 (professional* or formal* or external* or official*) N4 regulat*)) 11

S29 S18 OR S19 OR S20 OR S21 OR S22 OR S23 OR S24 OR S25 OR S26 OR S27 OR S28 3,837

S30 S17 OR S29 3,926

S31 (MH "Professional Practice") 14,752

S32 (MH "Practice Patterns") 12,163

S33 (MH "Professional Autonomy") 4,520

S34 S31 OR S32 OR S33 31,070

S35 S15 AND S34 844

S36 TI (practice* N6 (legal* or liabilit* or complaint* or litigat* or claim* or lawsuit* or prosecut* or compensation* or damages or "being sued" or malpractice or negligen* or misconduct)) OR AB (practice* N6 (legal* or liabilit* or complaint* or litigat* or claim* or lawsuit* or prosecut* or compensation* or damages or "being sued" or malpractice or negligen* or misconduct)) 3,338

S37 TI (behav* N6 (legal* or liabilit* or complaint* or litigat* or claim* or lawsuit* or prosecut* or compensation* or damages or "being sued" or malpractice or negligen* or misconduct)) OR AB (behav* N6 (legal* or liabilit* or complaint* or litigat* or claim* or lawsuit* or prosecut* or compensation* or damages or "being sued" or malpractice or negligen* or misconduct)) 1,361

S38 TI (autonom* N6 (legal* or liabilit* or complaint* or litigat* or claim* or lawsuit* or prosecut* or compensation* or damages or "being sued" or malpractice or negligen* or misconduct)) OR AB (autonom* N6 (legal* or liabilit* or complaint* or litigat* or claim* or lawsuit* or prosecut* or compensation* or damages or "being sued" or malpractice or negligen* or misconduct)) 329

S39	TI ((professional* or formal* or external* or official*) N6 regulat* N6 (practice* or behav* or autonom*)) OR AB ((professional* or formal* or external* or official*) N6 regulat* N6 (practice* or behav* or autonom*))	304
S40	TI (disciplinary N6 (action* or measure or measures or procedure* or proceeding* or process* or sanction*) N6 (practice* or behav* or autonom*)) OR AB (disciplinary N6 (action* or measure or measures or procedure* or proceeding* or process* or sanction*) N6 (practice* or behav* or autonom*))	64
S41	S36 OR S37 OR S38 OR S39 OR S40	5,323
S42	S35 OR S41	6,078
S43	TI (defensive* N3 (act or acts or action* or approach* or strateg*)) OR AB (defensive* N3 (act or acts or action* or approach* or strateg*))	136
S44	TI (legal* or liabilit* or complaint* or litigat* or claim* or lawsuit* or prosecut* or compensation* compensation* or damages or "being sued" or malpractice or negligen* or misconduct or regulat* or disciplin*) OR AB (legal* or liabilit* or complaint* or litigat* or claim* or lawsuit* or prosecut* or compensation* compensation* or damages or "being sued" or malpractice or negligen* or misconduct or regulat* or disciplin*)	282,678
S45	S15 OR S44	309,590
S46	S43 AND S45	23
S47	S5 OR S30 OR S42 OR S46	9,957
S48	(MH "Qualitative Studies+")	133,616
S49	(MH "Qualitative Validity+")	1,563
S50	(MH "Interviews+")	202,572
S51	(MH "Focus Groups")	39,562
S52	TI qualitative OR AB qualitative	111,074
S53	TI interview* OR AB interview*	189,113
S54	TI experience* OR AB experience*	364,137
S55	TI Focus N1 group* OR AB Focus N1 group*	31,393
S56	TI (attitude* or belief* or believ* or opinion* or perceiv* or perception* or preference* or view or views or viewpoint*) OR AB (attitude* or belief* or believ* or opinion* or perceiv* or perception* or preference* or view or views or viewpoint*)	420,614
S57	S48 OR S49 OR S50 OR S51 OR S52 OR S53 OR S54 OR S55 OR S56	904,477
S58	S47 AND S57	2,805
S59	(MH "Multimethod Studies")	13,122
S60	TI ((mixed N1 method* or multimethod* or multi N1 method*)) OR AB ((mixed N1 method* or multimethod* or multi N1 method*))	17,171
S61	(MH "Meta Synthesis")	1,465
S62	TI realist N1 synthes* OR AB realist N1 synthes*	124
S63	TI (meta-synthes* or metasynthes*) OR AB (meta-synthes* or metasynthes*)	1,027
S64	TI (meta-ethnograph* or metaethnograph*) OR AB (meta-ethnograph* or metaethnograph*)	425
S65	TI (meta-study or metastudy) OR AB (meta-study or metastudy)	81
S66	TI realist N1 review* OR AB realist N1 review*	207
S67	S59 OR S60 OR S61 OR S62 OR S63 OR S64 OR S65 OR S66	27,364
S68	S47 AND S67	65
S69	S58 OR S68	2,816
S70	S58 OR S68 Limiters - Published Date: 20000101-20201231	2,559

ASSIA

via ProQuest https://www.proquest.com/

1987 to current

Searched on: 7th January 2020

Records retrieved: 1246

Due to the limited functionality of the search interface the search strategy had to be split into 11 search lines and the results for each line downloaded into an EndNote library for deduplication.

S1	(TI,AB,IF(defensive* NEAR/4 (medicine OR medical)) OR TI,AB,IF(defensive* NEAR/4 practic*) OR TI,AB,IF(defensive* NEAR/4	57

	decision*) OR TI,AB,IF(defensive* NEAR/4 work*)) AND (((MAINSUBJECT.EXACT("Qualitative data") OR MAINSUBJECT.EXACT("Qualitative methods") OR MAINSUBJECT.EXACT("Qualitative analysis") OR MAINSUBJECT.EXACT("Qualitative research")) OR su(interview*) OR MAINSUBJECT.EXACT("Focus groups")) OR (TI,AB,IF,SU(qualitative OR interview* OR experience*) OR TI,AB,IF,SU(Focus NEAR/1 group*) OR TI,AB(attitude* OR belief* OR believ* OR opinion* OR perceiv* OR perception* OR preference* OR view OR views OR viewpoint*)) OR (MAINSUBJECT.EXACT("Multimethod research") OR (TI,AB,IF,SU(mixed NEAR/1 method* OR multimethod* OR multi NEAR/1 method*) OR TI,AB,IF,SU(realist NEAR/1 synthes*) OR TI,AB,IF,SU(meta-synthes* OR metasynthes*) OR TI,AB,IF,SU(meta-ethnograph* OR metaethnograph*) OR TI,AB,IF,SU(meta-study OR metastudy) OR TI,AB,IF,SU(realist NEAR/1 review*)))) AND pd(2000-2020)	
S2	(MAINSUBJECT.EXACT("Fear") AND ((MAINSUBJECT.EXACT.EXPLODE("Liability") OR MAINSUBJECT.EXACT("Jurisprudence") OR MAINSUBJECT.EXACT("Medical malpractice") OR MAINSUBJECT.EXACT("Professional misconduct") OR SU(malpractice) OR MAINSUBJECT.EXACT("Disciplinary procedures") OR MAINSUBJECT.EXACT.EXPLODE("Compensation") OR MAINSUBJECT.EXACT.EXPLODE("Damages") OR MAINSUBJECT.EXACT("Litigation") OR MAINSUBJECT.EXACT("Claims")) OR ((MAINSUBJECT.EXACT("Negligence") OR MAINSUBJECT.EXACT("Medical negligence")) OR MAINSUBJECT.EXACT("Regulation")))) AND (((MAINSUBJECT.EXACT("Qualitative data") OR MAINSUBJECT.EXACT("Qualitative methods") OR MAINSUBJECT.EXACT("Qualitative analysis") OR MAINSUBJECT.EXACT("Qualitative research")) OR su(interview*) OR MAINSUBJECT.EXACT("Focus groups")) OR (TI,AB,IF,SU(qualitative OR interview* OR experience*) OR TI,AB,IF,SU(Focus NEAR/1 group*) OR TI,AB(attitude* OR belief* OR believ* OR opinion* OR perceiv* OR perception* OR preference* OR view OR views OR viewpoint*)) OR (MAINSUBJECT.EXACT("Multimethod research") OR (TI,AB,IF,SU(mixed NEAR/1 method* OR multimethod* OR multi NEAR/1 method*) OR TI,AB,IF,SU(realist NEAR/1 synthes*) OR TI,AB,IF,SU(meta-synthes* OR metasynthes*) OR TI,AB,IF,SU(meta-ethnograph* OR metaethnograph*) OR TI,AB,IF,SU(meta-study OR metastudy) OR TI,AB,IF,SU(realist NEAR/1 review*)))) AND pd(2000-2020)	6
S3	TI,AB((fear* OR anxiet* OR worry OR worrie* OR apprehensi*) NEAR/4 (legal* OR liabilit* OR complaint* OR litigat* OR claim* OR lawsuit* OR prosecut* OR compensation* OR damages OR "being sued" OR malpractice OR negligen* OR misconduct)) AND (((MAINSUBJECT.EXACT("Qualitative data") OR MAINSUBJECT.EXACT("Qualitative methods") OR MAINSUBJECT.EXACT("Qualitative analysis") OR MAINSUBJECT.EXACT("Qualitative research")) OR su(interview*) OR MAINSUBJECT.EXACT("Focus groups")) OR (TI,AB,IF,SU(qualitative OR interview* OR experience*) OR TI,AB,IF,SU(Focus NEAR/1 group*) OR TI,AB(attitude* OR belief* OR believ* OR opinion* OR perceiv* OR perception* OR preference* OR view OR views OR viewpoint*)) OR (MAINSUBJECT.EXACT("Multimethod research") OR (TI,AB,IF,SU(mixed NEAR/1 method* OR multimethod* OR multi NEAR/1 method*) OR TI,AB,IF,SU(realist NEAR/1 synthes*) OR TI,AB,IF,SU(meta-synthes* OR metasynthes*) OR TI,AB,IF,SU(meta-ethnograph* OR metaethnograph*) OR TI,AB,IF,SU(meta-study OR metastudy) OR TI,AB,IF,SU(realist NEAR/1 review*)))) AND pd(2000-2020)	187

S4	TI,AB((afraid OR dread* OR threat* OR expos* OR avoid*) NEAR/4 (legal* OR liabilit* OR complaint* OR litigat* OR claim* OR lawsuit* OR prosecut* OR compensation* OR damages OR "being sued" OR malpractice OR negligen* OR misconduct)) AND (((MAINSUBJECT.EXACT("Qualitative data") OR MAINSUBJECT.EXACT("Qualitative methods") OR MAINSUBJECT.EXACT("Qualitative analysis") OR MAINSUBJECT.EXACT("Qualitative research")) OR su(interview*) OR MAINSUBJECT.EXACT("Focus groups")) OR (TI,AB,IF,SU(qualitative OR interview* OR experience*) OR TI,AB,IF,SU(Focus NEAR/1 group*) OR TI,AB(attitude* OR belief* OR believ* OR opinion* OR perceiv* OR perception* OR preference* OR view OR views OR viewpoint*)) OR (MAINSUBJECT.EXACT("Multimethod research") OR (TI,AB,IF,SU(mixed NEAR/1 method* OR multimethod* OR multi NEAR/1 method*) OR TI,AB,IF,SU(realist NEAR/1 synthes*) OR TI,AB,IF,SU(meta-synthes* OR metasynthes*) OR TI,AB,IF,SU(meta-ethnograph* OR metaethnograph*) OR TI,AB,IF,SU(meta-study OR metastudy) OR TI,AB,IF,SU(realist NEAR/1 review*)))) AND pd(2000-2020)	157
S5	TI,AB((fear* OR anxiet* OR worry OR worrie* OR apprehensi* OR afraid OR dread* OR threat* OR expos* OR avoid*) NEAR/4 disciplin* NEAR/4 (action* OR measure OR measures OR procedure* OR proceeding* OR process* OR sanction*)) AND (((MAINSUBJECT.EXACT("Qualitative data") OR MAINSUBJECT.EXACT("Qualitative methods") OR MAINSUBJECT.EXACT("Qualitative analysis") OR MAINSUBJECT.EXACT("Qualitative research")) OR su(interview*) OR MAINSUBJECT.EXACT("Focus groups")) OR (TI,AB,IF,SU(qualitative OR interview* OR experience*) OR TI,AB,IF,SU(Focus NEAR/1 group*) OR TI,AB(attitude* OR belief* OR believ* OR opinion* OR perceiv* OR perception* OR preference* OR view OR views OR viewpoint*)) OR (MAINSUBJECT.EXACT("Multimethod research") OR (TI,AB,IF,SU(mixed NEAR/1 method* OR multimethod* OR multi NEAR/1 method*) OR TI,AB,IF,SU(realist NEAR/1 synthes*) OR TI,AB,IF,SU(meta-synthes* OR metasynthes*) OR TI,AB,IF,SU(meta-ethnograph* OR metaethnograph*) OR TI,AB,IF,SU(meta-study OR metastudy) OR TI,AB,IF,SU(realist NEAR/1 review*)))) AND pd(2000-2020)	3
S6	TI,AB((fear* OR anxiet* OR worry OR worrie* OR apprehensi* OR afraid OR dread* OR threat* OR expos* OR avoid*) NEAR/4 (professional* OR formal* OR external* OR official*) NEAR/4 regulat*) AND (((MAINSUBJECT.EXACT("Qualitative data") OR MAINSUBJECT.EXACT("Qualitative methods") OR MAINSUBJECT.EXACT("Qualitative analysis") OR MAINSUBJECT.EXACT("Qualitative research")) OR su(interview*) OR MAINSUBJECT.EXACT("Focus groups")) OR (TI,AB,IF,SU(qualitative OR interview* OR experience*) OR TI,AB,IF,SU(Focus NEAR/1 group*) OR TI,AB(attitude* OR belief* OR believ* OR opinion* OR perceiv* OR perception* OR preference* OR view OR views OR viewpoint*)) OR (MAINSUBJECT.EXACT("Multimethod research") OR (TI,AB,IF,SU(mixed NEAR/1 method* OR multimethod* OR multi NEAR/1 method*) OR TI,AB,IF,SU(realist NEAR/1 synthes*) OR TI,AB,IF,SU(meta-synthes* OR metasynthes*) OR TI,AB,IF,SU(meta-ethnograph* OR metaethnograph*) OR TI,AB,IF,SU(meta-study OR metastudy) OR TI,AB,IF,SU(realist NEAR/1 review*)))) AND pd(2000-2020)	1
S7	TI,AB((practice* OR behav* OR autonom*) NEAR/6 (legal* OR liabilit* OR complaint* OR litigat* OR claim* OR lawsuit* OR prosecut* OR compensation* OR damages OR "being sued" OR malpractice OR negligen* OR misconduct)) AND (((MAINSUBJECT.EXACT("Qualitative data") OR MAINSUBJECT.EXACT("Qualitative methods") OR MAINSUBJECT.EXACT("Qualitative analysis") OR MAINSUBJECT.EXACT("Qualitative research")) OR su(interview*) OR	705

	MAINSUBJECT.EXACT("Focus groups")) OR (TI,AB,IF,SU(qualitative OR interview* OR experience*) OR TI,AB,IF,SU(Focus NEAR/1 group*) OR TI,AB(attitude* OR belief* OR believ* OR opinion* OR perceiv* OR perception* OR preference* OR view OR views OR viewpoint*)) OR (MAINSUBJECT.EXACT("Multimethod research") OR (TI,AB,IF,SU(mixed NEAR/1 method* OR multimethod* OR multi NEAR/1 method*) OR TI,AB,IF,SU(realist NEAR/1 synthes*) OR TI,AB,IF,SU(meta-synthes* OR metasynthes*) OR TI,AB,IF,SU(meta-ethnograph* OR metaethnograph*) OR TI,AB,IF,SU(meta-study OR metastudy) OR TI,AB,IF,SU(realist NEAR/1 review*)))) AND pd(2000-2020)	
S8	TI,AB((professional* OR formal* OR external* OR official*) NEAR/6 regulat* NEAR/6 (practice* OR behav* OR autonom*)) AND (((MAINSUBJECT.EXACT("Qualitative data") OR MAINSUBJECT.EXACT("Qualitative methods") OR MAINSUBJECT.EXACT("Qualitative analysis") OR MAINSUBJECT.EXACT("Qualitative research")) OR su(interview*) OR MAINSUBJECT.EXACT("Focus groups")) OR (TI,AB,IF,SU(qualitative OR interview* OR experience*) OR TI,AB,IF,SU(Focus NEAR/1 group*) OR TI,AB(attitude* OR belief* OR believ* OR opinion* OR perceiv* OR perception* OR preference* OR view OR views OR viewpoint*)) OR (MAINSUBJECT.EXACT("Multimethod research") OR (TI,AB,IF,SU(mixed NEAR/1 method* OR multimethod* OR multi NEAR/1 method*) OR TI,AB,IF,SU(realist NEAR/1 synthes*) OR TI,AB,IF,SU(meta-synthes* OR metasynthes*) OR TI,AB,IF,SU(meta-ethnograph* OR metaethnograph*) OR TI,AB,IF,SU(meta-study OR metastudy) OR TI,AB,IF,SU(realist NEAR/1 review*)))) AND pd(2000-2020)	55
S9	TI,AB(disciplinary NEAR/6 (action* OR measure OR measures OR procedure* OR proceeding* OR process* OR sanction*) NEAR/6 (practice* OR behav* OR autonom*)) AND (((MAINSUBJECT.EXACT("Qualitative data") OR MAINSUBJECT.EXACT("Qualitative methods") OR MAINSUBJECT.EXACT("Qualitative analysis") OR MAINSUBJECT.EXACT("Qualitative research")) OR su(interview*) OR MAINSUBJECT.EXACT("Focus groups")) OR (TI,AB,IF,SU(qualitative OR interview* OR experience*) OR TI,AB,IF,SU(Focus NEAR/1 group*) OR TI,AB(attitude* OR belief* OR believ* OR opinion* OR perceiv* OR perception* OR preference* OR view OR views OR viewpoint*)) OR (MAINSUBJECT.EXACT("Multimethod research") OR (TI,AB,IF,SU(mixed NEAR/1 method* OR multimethod* OR multi NEAR/1 method*) OR TI,AB,IF,SU(realist NEAR/1 synthes*) OR TI,AB,IF,SU(meta-synthes* OR metasynthes*) OR TI,AB,IF,SU(meta-ethnograph* OR metaethnograph*) OR TI,AB,IF,SU(meta-study OR metastudy) OR TI,AB,IF,SU(realist NEAR/1 review*)))) AND pd(2000-2020)	17
S10	((((MAINSUBJECT.EXACT.EXPLODE("Liability") OR MAINSUBJECT.EXACT("Jurisprudence") OR MAINSUBJECT.EXACT("Medical malpractice") OR MAINSUBJECT.EXACT("Professional misconduct") OR SU(malpractice) OR MAINSUBJECT.EXACT("Disciplinary procedures") OR MAINSUBJECT.EXACT.EXPLODE("Compensation") OR MAINSUBJECT.EXACT.EXPLODE("Damages") OR MAINSUBJECT.EXACT("Litigation") OR MAINSUBJECT.EXACT("Claims")) OR ((MAINSUBJECT.EXACT("Negligence") OR MAINSUBJECT.EXACT("Medical negligence")) OR MAINSUBJECT.EXACT("Regulation"))) OR TI,AB(legal* OR liabilit* OR complaint* OR litigat* OR claim* OR lawsuit* OR prosecut* OR compensation* compensation* OR damages OR "being sued" OR malpractice OR negligen* OR misconduct OR regulat* OR disciplin*)) AND TI,AB(defensive* NEAR/3 (act OR acts OR action* OR approach* OR strateg*))) AND (((MAINSUBJECT.EXACT("Qualitative data") OR	8

	MAINSUBJECT.EXACT("Qualitative methods") OR MAINSUBJECT.EXACT("Qualitative analysis") OR MAINSUBJECT.EXACT("Qualitative research")) OR su(interview*) OR MAINSUBJECT.EXACT("Focus groups")) OR (TI,AB,IF,SU(qualitative OR interview* OR experience*) OR TI,AB,IF,SU(Focus NEAR/1 group*) OR TI,AB(attitude* OR belief* OR believ* OR opinion* OR perceiv* OR perception* OR preference* OR view OR views OR viewpoint*)) OR (MAINSUBJECT.EXACT("Multimethod research") OR (TI,AB,IF,SU(mixed NEAR/1 method* OR multimethod* OR multi NEAR/1 method*) OR TI,AB,IF,SU(realist NEAR/1 synthes*) OR TI,AB,IF,SU(meta-synthes* OR metasynthes*) OR TI,AB,IF,SU(meta-ethnograph* OR metaethnograph*) OR TI,AB,IF,SU(meta-study OR metastudy) OR TI,AB,IF,SU(realist NEAR/1 review*)))) AND pd(2000-2020)	
S11	((MAINSUBJECT.EXACT("Professional practices") OR MAINSUBJECT.EXACT("Practice") OR MAINSUBJECT.EXACT("Autonomous practice") OR MAINSUBJECT.EXACT("Professional autonomy") OR MAINSUBJECT.EXACT("Autonomy")) AND ((MAINSUBJECT.EXACT.EXPLODE("Liability") OR MAINSUBJECT.EXACT("Jurisprudence") OR MAINSUBJECT.EXACT("Medical malpractice") OR MAINSUBJECT.EXACT("Professional misconduct") OR SU(malpractice) OR MAINSUBJECT.EXACT("Disciplinary procedures") OR MAINSUBJECT.EXACT.EXPLODE("Compensation") OR MAINSUBJECT.EXACT.EXPLODE("Damages") OR MAINSUBJECT.EXACT("Litigation") OR MAINSUBJECT.EXACT("Claims")) OR ((MAINSUBJECT.EXACT("Negligence") OR MAINSUBJECT.EXACT("Medical negligence")) OR MAINSUBJECT.EXACT("Regulation")))) AND (((MAINSUBJECT.EXACT("Qualitative data") OR MAINSUBJECT.EXACT("Qualitative methods") OR MAINSUBJECT.EXACT("Qualitative analysis") OR MAINSUBJECT.EXACT("Qualitative research")) OR su(interview*) OR MAINSUBJECT.EXACT("Focus groups")) OR (TI,AB,IF,SU(qualitative OR interview* OR experience*) OR TI,AB,IF,SU(Focus NEAR/1 group*) OR TI,AB(attitude* OR belief* OR believ* OR opinion* OR perceiv* OR perception* OR preference* OR view OR views OR viewpoint*)) OR (MAINSUBJECT.EXACT("Multimethod research") OR (TI,AB,IF,SU(mixed NEAR/1 method* OR multimethod* OR multi NEAR/1 method*) OR TI,AB,IF,SU(realist NEAR/1 synthes*) OR TI,AB,IF,SU(meta-synthes* OR metasynthes*) OR TI,AB,IF,SU(meta-ethnograph* OR metaethnograph*) OR TI,AB,IF,SU(meta-study OR metastudy) OR TI,AB,IF,SU(realist NEAR/1 review*)))) AND pd(2000-2020)	50

Sociological Abstracts
via ProQuest https://www.proquest.com/
1952 to current
Searched on: 8th January 2020
Records retrieved: 1916

Due to the limited functionality of the search interface the search strategy had to be split into 11 search lines and the results for each line downloaded into an EndNote library for deduplication.

S1	(TI,AB,IF(defensive* NEAR/4 (medicine OR medical)) OR TI,AB,IF(defensive* NEAR/4 practic*) OR TI,AB,IF(defensive* NEAR/4 decision*) OR TI,AB,IF(defensive* NEAR/4 work*)) AND ((MAINSUBJECT.EXACT("Qualitative Methods") OR	33

	MAINSUBJECT.EXACT("Interviews") OR MAINSUBJECT.EXACT("Group Research") OR TI,AB,IF,SU(qualitative) OR TI,AB,IF,SU(Interview*) OR TI,AB,IF,SU(Experience*) OR TI,AB,IF,SU(Focus NEAR/1 group*) OR TI,AB(attitude* OR belief* OR believ* OR opinion* OR perceiv* OR perception* OR preference* OR view OR views OR viewpoint*)) OR (TI,AB,IF,SU(mixed NEAR/1 method* OR multimethod* OR multi NEAR/1 method*) OR TI,AB,IF,SU(realist NEAR/1 synthes*) OR TI,AB,IF,SU(meta-synthes* OR metasynthes*) OR TI,AB,IF,SU(meta-ethnograph* OR metaethnograph*) OR TI,AB,IF,SU(meta-study OR metastudy) OR TI,AB,IF,SU(realist NEAR/1 review*))) AND pd(2000-2020)	
S2	((MAINSUBJECT.EXACT("Liability") OR su(("Professional liability")) OR MAINSUBJECT.EXACT("Jurisprudence") OR MAINSUBJECT.EXACT("Legal Procedure") OR MAINSUBJECT.EXACT("Professional Malpractice") OR su(malpractice) OR su("Medical malpractice") OR su("Professional misconduct") OR su("employee discipline") OR MAINSUBJECT.EXACT("Compensation") OR MAINSUBJECT.EXACT("Litigation") OR MAINSUBJECT.EXACT("Regulation")) AND MAINSUBJECT.EXACT("Fear")) AND ((MAINSUBJECT.EXACT("Qualitative Methods") OR MAINSUBJECT.EXACT("Interviews") OR MAINSUBJECT.EXACT("Group Research") OR TI,AB,IF,SU(qualitative) OR TI,AB,IF,SU(Interview*) OR TI,AB,IF,SU(Experience*) OR TI,AB,IF,SU(Focus NEAR/1 group*) OR TI,AB(attitude* OR belief* OR believ* OR opinion* OR perceiv* OR perception* OR preference* OR view OR views OR viewpoint*)) OR (TI,AB,IF,SU(mixed NEAR/1 method* OR multimethod* OR multi NEAR/1 method*) OR TI,AB,IF,SU(realist NEAR/1 synthes*) OR TI,AB,IF,SU(meta-synthes* OR metasynthes*) OR TI,AB,IF,SU(meta-ethnograph* OR metaethnograph*) OR TI,AB,IF,SU(meta-study OR metastudy) OR TI,AB,IF,SU(realist NEAR/1 review*))) AND pd(2000-2020)	14
S4	((MAINSUBJECT.EXACT("Qualitative Methods") OR MAINSUBJECT.EXACT("Interviews") OR MAINSUBJECT.EXACT("Group Research") OR TI,AB,IF,SU(qualitative) OR TI,AB,IF,SU(Interview*) OR TI,AB,IF,SU(Experience*) OR TI,AB,IF,SU(Focus NEAR/1 group*) OR TI,AB(attitude* OR belief* OR believ* OR opinion* OR perceiv* OR perception* OR preference* OR view OR views OR viewpoint*)) OR (TI,AB,IF,SU(mixed NEAR/1 method* OR multimethod* OR multi NEAR/1 method*) OR TI,AB,IF,SU(realist NEAR/1 synthes*) OR TI,AB,IF,SU(meta-synthes* OR metasynthes*) OR TI,AB,IF,SU(meta-ethnograph* OR metaethnograph*) OR TI,AB,IF,SU(meta-study OR metastudy) OR TI,AB,IF,SU(realist NEAR/1 review*))) AND TI,AB((fear* OR anxiet* OR worry OR worrie* OR apprehensi*) NEAR/4 (legal* OR liabilit* OR complaint* OR litigat* OR claim* OR lawsuit* OR prosecut* OR compensation* OR damages OR "being sued" OR malpractice OR negligen* OR misconduct)) AND pd(2000-2020)	103
S5	((MAINSUBJECT.EXACT("Qualitative Methods") OR MAINSUBJECT.EXACT("Interviews") OR MAINSUBJECT.EXACT("Group Research") OR TI,AB,IF,SU(qualitative) OR TI,AB,IF,SU(Interview*) OR TI,AB,IF,SU(Experience*) OR TI,AB,IF,SU(Focus NEAR/1 group*) OR TI,AB(attitude* OR belief* OR believ* OR opinion* OR perceiv* OR perception* OR preference* OR view OR views OR viewpoint*)) OR (TI,AB,IF,SU(mixed NEAR/1 method* OR multimethod* OR multi NEAR/1 method*) OR TI,AB,IF,SU(realist NEAR/1 synthes*) OR TI,AB,IF,SU(meta-synthes* OR metasynthes*) OR TI,AB,IF,SU(meta-ethnograph* OR metaethnograph*) OR TI,AB,IF,SU(meta-study OR metastudy) OR TI,AB,IF,SU(realist NEAR/1 review*))) AND TI,AB((afraid OR dread* OR threat* OR expos* OR avoid*) NEAR/4 (legal* OR liabilit* OR complaint* OR litigat* OR claim* OR lawsuit* OR prosecut* OR compensation* OR damages	300

	OR "being sued" OR malpractice OR negligen* OR misconduct)) AND pd(2000-2020)	
S6	((MAINSUBJECT.EXACT("Qualitative Methods") OR MAINSUBJECT.EXACT("Interviews") OR MAINSUBJECT.EXACT("Group Research") OR TI,AB,IF,SU(qualitative) OR TI,AB,IF,SU(Interview*) OR TI,AB,IF,SU(Experience*) OR TI,AB,IF,SU(Focus NEAR/1 group*) OR TI,AB(attitude* OR belief* OR believ* OR opinion* OR perceiv* OR perception* OR preference* OR view OR views OR viewpoint*)) OR (TI,AB,IF,SU(mixed NEAR/1 method* OR multimethod* OR multi NEAR/1 method*) OR TI,AB,IF,SU(realist NEAR/1 synthes*) OR TI,AB,IF,SU(meta-synthes* OR metasynthes*) OR TI,AB,IF,SU(meta-ethnograph* OR metaethnograph*) OR TI,AB,IF,SU(meta-study OR metastudy) OR TI,AB,IF,SU(realist NEAR/1 review*))) AND TI,AB((fear* OR anxiet* OR worry OR worrie* OR apprehensi* OR afraid OR dread* OR threat* OR expos* OR avoid*) NEAR/4 disciplin* NEAR/4 (action* OR measure OR measures OR procedure* OR proceeding* OR process* OR sanction*)) AND pd(2000-2020)	6
S7	((MAINSUBJECT.EXACT("Qualitative Methods") OR MAINSUBJECT.EXACT("Interviews") OR MAINSUBJECT.EXACT("Group Research") OR TI,AB,IF,SU(qualitative) OR TI,AB,IF,SU(Interview*) OR TI,AB,IF,SU(Experience*) OR TI,AB,IF,SU(Focus NEAR/1 group*) OR TI,AB(attitude* OR belief* OR believ* OR opinion* OR perceiv* OR perception* OR preference* OR view OR views OR viewpoint*)) OR (TI,AB,IF,SU(mixed NEAR/1 method* OR multimethod* OR multi NEAR/1 method*) OR TI,AB,IF,SU(realist NEAR/1 synthes*) OR TI,AB,IF,SU(meta-synthes* OR metasynthes*) OR TI,AB,IF,SU(meta-ethnograph* OR metaethnograph*) OR TI,AB,IF,SU(meta-study OR metastudy) OR TI,AB,IF,SU(realist NEAR/1 review*))) AND TI,AB((fear* OR anxiet* OR worry OR worrie* OR apprehensi* OR afraid OR dread* OR threat* OR expos* OR avoid*) NEAR/4 (professional* OR formal* OR external* OR official*) NEAR/4 regulat*) AND pd(2000-2020)	3
S8	((MAINSUBJECT.EXACT("Qualitative Methods") OR MAINSUBJECT.EXACT("Interviews") OR MAINSUBJECT.EXACT("Group Research") OR TI,AB,IF,SU(qualitative) OR TI,AB,IF,SU(Interview*) OR TI,AB,IF,SU(Experience*) OR TI,AB,IF,SU(Focus NEAR/1 group*) OR TI,AB(attitude* OR belief* OR believ* OR opinion* OR perceiv* OR perception* OR preference* OR view OR views OR viewpoint*)) OR (TI,AB,IF,SU(mixed NEAR/1 method* OR multimethod* OR multi NEAR/1 method*) OR TI,AB,IF,SU(realist NEAR/1 synthes*) OR TI,AB,IF,SU(meta-synthes* OR metasynthes*) OR TI,AB,IF,SU(meta-ethnograph* OR metaethnograph*) OR TI,AB,IF,SU(meta-study OR metastudy) OR TI,AB,IF,SU(realist NEAR/1 review*))) AND TI,AB((practice* OR behav* OR autonom*) NEAR/6 (legal* OR liabilit* OR complaint* OR litigat* OR claim* OR lawsuit* OR prosecut* OR compensation* OR damages OR "being sued" OR malpractice OR negligen* OR misconduct)) AND pd(2000-2020)	1384
S9	((MAINSUBJECT.EXACT("Qualitative Methods") OR MAINSUBJECT.EXACT("Interviews") OR MAINSUBJECT.EXACT("Group Research") OR TI,AB,IF,SU(qualitative) OR TI,AB,IF,SU(Interview*) OR TI,AB,IF,SU(Experience*) OR TI,AB,IF,SU(Focus NEAR/1 group*) OR TI,AB(attitude* OR belief* OR believ* OR opinion* OR perceiv* OR perception* OR preference* OR view OR views OR viewpoint*)) OR (TI,AB,IF,SU(mixed NEAR/1 method* OR multimethod* OR multi NEAR/1 method*) OR TI,AB,IF,SU(realist NEAR/1 synthes*) OR TI,AB,IF,SU(meta-synthes* OR metasynthes*) OR TI,AB,IF,SU(meta-ethnograph* OR metaethnograph*) OR TI,AB,IF,SU(meta-study OR metastudy) OR TI,AB,IF,SU(realist NEAR/1 review*))) AND TI,AB((professional* OR formal* OR external* OR official*) NEAR/6 regulat* NEAR/6 (practice* OR behav* OR autonom*)) AND pd(2000-2020)	54

S10	((MAINSUBJECT.EXACT("Qualitative Methods") OR MAINSUBJECT.EXACT("Interviews") OR MAINSUBJECT.EXACT("Group Research") OR TI,AB,IF,SU(qualitative) OR TI,AB,IF,SU(Interview*) OR TI,AB,IF,SU(Experience*) OR TI,AB,IF,SU(Focus NEAR/1 group*) OR TI,AB(attitude* OR belief* OR believ* OR opinion* OR perceiv* OR perception* OR preference* OR view OR views OR viewpoint*)) OR (TI,AB,IF,SU(mixed NEAR/1 method* OR multimethod* OR multi NEAR/1 method*) OR TI,AB,IF,SU(realist NEAR/1 synthes*) OR TI,AB,IF,SU(meta-synthes* OR metasynthes*) OR TI,AB,IF,SU(meta-ethnograph* OR metaethnograph*) OR TI,AB,IF,SU(meta-study OR metastudy) OR TI,AB,IF,SU(realist NEAR/1 review*))) AND TI,AB(disciplinary NEAR/6 (action* OR measure OR measures OR procedure* OR proceeding* OR process* OR sanction*) NEAR/6 (practice* OR behav* OR autonom*)) AND pd(2000-2020)	14
S11	(TI,AB(defensive* NEAR/3 (act OR acts OR action* OR approach* OR strateg*)) AND ((MAINSUBJECT.EXACT("Liability") OR su(("Professional liability")) OR MAINSUBJECT.EXACT("Jurisprudence") OR MAINSUBJECT.EXACT("Legal Procedure") OR MAINSUBJECT.EXACT("Professional Malpractice") OR su(malpractice) OR su("Medical malpractice") OR su("Professional misconduct") OR su("employee discipline") OR MAINSUBJECT.EXACT("Compensation") OR MAINSUBJECT.EXACT("Litigation") OR MAINSUBJECT.EXACT("Regulation")) OR TI,AB(legal* OR liabilit* OR complaint* OR litigat* OR claim* OR lawsuit* OR prosecut* OR compensation* compensation* OR damages OR "being sued" OR malpractice OR negligen* OR misconduct OR regulat* OR disciplin*))) AND ((MAINSUBJECT.EXACT("Qualitative Methods") OR MAINSUBJECT.EXACT("Interviews") OR MAINSUBJECT.EXACT("Group Research") OR TI,AB,IF,SU(qualitative) OR TI,AB,IF,SU(Interview*) OR TI,AB,IF,SU(Experience*) OR TI,AB,IF,SU(Focus NEAR/1 group*) OR TI,AB(attitude* OR belief* OR believ* OR opinion* OR perceiv* OR perception* OR preference* OR view OR views OR viewpoint*)) OR (TI,AB,IF,SU(mixed NEAR/1 method* OR multimethod* OR multi NEAR/1 method*) OR TI,AB,IF,SU(realist NEAR/1 synthes*) OR TI,AB,IF,SU(meta-synthes* OR metasynthes*) OR TI,AB,IF,SU(meta-ethnograph* OR metaethnograph*) OR TI,AB,IF,SU(meta-study OR metastudy) OR TI,AB,IF,SU(realist NEAR/1 review*))) AND pd(2000-2020)	13

ProQuest Dissertations & Theses A&I
via ProQuest https://www.proquest.com/
Inception to current
Searched on: 8th January 2020
Records retrieved: 400

Due to the limited functionality of the search interface a pragmatic approach was taken, limiting the search to key terms only. Six search lines were used with the results for each line downloaded into an EndNote library for deduplication

S1	(TI,AB,IF,SU(defensive* NEAR/4 (medicine OR medical)) OR TI,AB,IF,SU(defensive* NEAR/4 practic*) OR TI,AB,IF,SU(defensive* NEAR/4 decision*) OR TI,AB,IF,SU(defensive* NEAR/4 work*)) AND (TI,AB,IF,SU(qualitative OR interview* OR experience*) OR TI,AB,IF,SU(Focus NEAR/1 group*) OR TI,AB(attitude* OR belief* OR believ* OR opinion* OR perceiv* OR perception* OR preference* OR view OR views OR viewpoint*) OR TI,AB,IF,SU(mixed NEAR/1 method* OR multimethod* OR multi NEAR/1 method*) OR TI,AB,IF,SU(realist NEAR/1 synthes*) OR TI,AB,IF,SU(meta-synthes* OR metasynthes*) OR	86

	TI,AB,IF,SU(meta-ethnograph* OR metaethnograph*) OR TI,AB,IF,SU(meta-study OR metastudy) OR TI,AB,IF,SU(realist NEAR/1 review*)) AND pd(20000101-20201231)	
S2	TI,AB,IF,SU(fear* NEAR/4 (legal* OR liabilit* OR complaint* OR litigat* OR claim* OR lawsuit* OR prosecut* OR compensation* OR damages OR "being sued" OR malpractice OR negligen* OR misconduct)) AND (TI,AB,IF,SU(qualitative OR interview* OR experience*) OR TI,AB,IF,SU(Focus NEAR/1 group*) OR TI,AB(attitude* OR belief* OR believ* OR opinion* OR perceiv* OR perception* OR preference* OR view OR views OR viewpoint*) OR TI,AB,IF,SU(mixed NEAR/1 method* OR multimethod* OR multi NEAR/1 method*) OR TI,AB,IF,SU(realist NEAR/1 synthes*) OR TI,AB,IF,SU(meta-synthes* OR metasynthes*) OR TI,AB,IF,SU(meta-ethnograph* OR metaethnograph*) OR TI,AB,IF,SU(meta-study OR metastudy) OR TI,AB,IF,SU(realist NEAR/1 review*)) AND pd(20000101-20201231)	123
S3	TI,AB,IF,SU((fear* OR anxiet* OR worry OR worrie* OR apprehensi* OR afraid OR dread* OR threat* OR expos* OR avoid*) NEAR/4 (professional* OR formal* OR external* OR official*) NEAR/4 regulat*) AND (TI,AB,IF,SU(qualitative OR interview* OR experience*) OR TI,AB,IF,SU(Focus NEAR/1 group*) OR TI,AB(attitude* OR belief* OR believ* OR opinion* OR perceiv* OR perception* OR preference* OR view OR views OR viewpoint*) OR TI,AB,IF,SU(mixed NEAR/1 method* OR multimethod* OR multi NEAR/1 method*) OR TI,AB,IF,SU(realist NEAR/1 synthes*) OR TI,AB,IF,SU(meta-synthes* OR metasynthes*) OR TI,AB,IF,SU(meta-ethnograph* OR metaethnograph*) OR TI,AB,IF,SU(meta-study OR metastudy) OR TI,AB,IF,SU(realist NEAR/1 review*)) AND pd(20000101-20201231)	8
S4	(TI,AB,IF,SU(qualitative OR interview* OR experience*) OR TI,AB,IF,SU(Focus NEAR/1 group*) OR TI,AB(attitude* OR belief* OR believ* OR opinion* OR perceiv* OR perception* OR preference* OR view OR views OR viewpoint*) OR TI,AB,IF,SU(mixed NEAR/1 method* OR multimethod* OR multi NEAR/1 method*) OR TI,AB,IF,SU(realist NEAR/1 synthes*) OR TI,AB,IF,SU(meta-synthes* OR metasynthes*) OR TI,AB,IF,SU(meta-ethnograph* OR metaethnograph*) OR TI,AB,IF,SU(meta-study OR metastudy) OR TI,AB,IF,SU(realist NEAR/1 review*)) AND TI,AB,IF,SU(defensive* NEAR/3 (act OR acts OR action* OR approach* OR strateg*)) AND pd(20000101-20201231)	173
S5	TI,AB,IF,SU((fear* OR anxiet* OR worry OR worrie* OR apprehensi* OR afraid OR dread* OR threat* OR expos* OR avoid*) NEAR/4 disciplin* NEAR/4 (action* OR measure OR measures OR procedure* OR proceeding* OR process* OR sanction*))	19
S6	TI,AB,IF,SU((fear* OR anxiet* OR worry OR worrie* OR apprehensi* OR afraid OR dread* OR threat* OR expos* OR avoid*) NEAR/4 disciplin* NEAR/4 (action* OR measure OR measures OR procedure* OR proceeding* OR process* OR sanction*)) AND pd(20000101-20201231)	10

PROSPERO
https://www.crd.york.ac.uk/prospero/
Searched on: 7th January 2020
Records retrieved: 62

#1	MeSH DESCRIPTOR Defensive medicine	0
#2	defensive* adj4 (medicine or medical)	0
#3	defensive* adj4 practic*	1
#4	defensive* adj4 decision*	0

#5 defensive* adj4 work* 0
#6 #1 OR #2 OR #3 OR #4 OR #5 1
#7 MeSH DESCRIPTOR Liability, Legal 1
#8 MeSH DESCRIPTOR Jurisprudence 2
#9 MeSH DESCRIPTOR Jurisprudence EXPLODE ALL TREES 130
#10 MeSH DESCRIPTOR Malpractice 2
#11 MeSH DESCRIPTOR Professional Misconduct 0
#12 MeSH DESCRIPTOR Professional Misconduct EXPLODE ALL TREES 3
#13 MeSH DESCRIPTOR Employee Discipline 0
#14 MeSH DESCRIPTOR Compensation and Redress 2
#15 #7 OR #8 OR #10 OR #11 OR #13 OR #146
#16 MeSH DESCRIPTOR Fear72
#17 #15 AND #16 0
#18 fear* adj4 (legal* or liabilit* or complaint* or litigat* or claim* or lawsuit* or prosecut* or compensation* or damages or "being sued" or malpractice or negligen* or misconduct) 2
#19 anxiet* adj4 (legal* or liabilit* or complaint* or litigat* or claim* or lawsuit* or prosecut* or compensation* or damages or "being sued" or malpractice or negligen* or misconduct) 10
#20 (worry or worrie*) adj4 (legal* or liabilit* or complaint* or litigat* or claim* or lawsuit* or prosecut* or compensation* or damages or "being sued" or malpractice or negligen* or misconduct) 0
#21 apprehensi* adj4 (legal* or liabilit* or complaint* or litigat* or claim* or lawsuit* or prosecut* or compensation* or damages or "being sued" or malpractice or negligen* or misconduct) 0
#22 afraid adj4 (legal* or liabilit* or complaint* or litigat* or claim* or lawsuit* or prosecut* or compensation* or damages or "being sued" or malpractice or negligen* or misconduct) 0
#23 dread* adj4 (legal* or liabilit* or complaint* or litigat* or claim* or lawsuit* or prosecut* or compensation* or damages or "being sued" or malpractice or negligen* or misconduct) 0
#24 threat* adj4 (legal* or liabilit* or complaint* or litigat* or claim* or lawsuit* or prosecut* or compensation* or damages or "being sued" or malpractice or negligen* or misconduct) 1
#25 expos* adj4 (legal* or liabilit* or complaint* or litigat* or claim* or lawsuit* or prosecut* or compensation* or damages or "being sued" or malpractice or negligen* or misconduct) 8
#26 avoid* adj4 (legal* or liabilit* or complaint* or litigat* or claim* or lawsuit* or prosecut* or compensation* or damages or "being sued" or malpractice or negligen* or misconduct) 1
#27 ((fear* or anxiet* or worry or worrie* or apprehensi* or afraid or dread* or threat* or expos* or avoid*) adj4 disciplin* adj4 (action* or measure or measures or procedure* or proceeding* or process* or sanction*)) 0
#28 ((fear* or anxiet* or worry or worrie* or apprehensi* or afraid or dread* or threat* or expos* or avoid*) adj4 (professional* or formal* or external* or official*) adj4 regulat*) 0
#29 #18 OR #19 OR #20 OR #21 OR #22 OR #23 OR #24 OR #25 OR #26 OR #27 OR #28 22
#30 #29 OR #17 22
#31 MeSH DESCRIPTOR Professional Practice 47
#32 MeSH DESCRIPTOR Practice Patterns, Physicians' 0
#33 MeSH DESCRIPTOR Practice Patterns, Dentists' 0
#34 MeSH DESCRIPTOR Institutional Practice 1
#35 MeSH DESCRIPTOR Professional Autonomy 5
#36 #31 OR #32 OR #33 OR #34 OR #35 51
#37 #36 AND #15 0
#38 practice* adj6 (legal* or liabilit* or complaint* or litigat* or claim* or lawsuit* or prosecut* or compensation* or damages or "being sued" or malpractice or negligen* or misconduct) 14
#39 behav* adj6 (legal* or liabilit* or complaint* or litigat* or claim* or lawsuit* or prosecut* or compensation* or damages or "being sued" or malpractice or negligen* or misconduct) 22

#40 autonom* adj6 (legal* or liabilit* or complaint* or litigat* or claim* or lawsuit* or prosecut* or compensation* or damages or "being sued" or malpractice or negligen* or misconduct) 2
#41 (professional* or formal* or external* or official*) adj6 regulat* adj6 (practice* or behav* or autonom*) 2
#42 disciplinary adj6 (action* or measure or measures or procedure* or proceeding* or process* or sanction*) adj6 (practice* or behav* or autonom*) 0
#43 #38 OR #39 OR #40 OR #41 OR #42 39
#44 #37 OR #43 39
#45 defensive* adj3 (act or acts or action* or approach* or strateg*)0
#46 #6 OR #30 OR #44 OR #45 62

Google Scholar

Two search strings were used in Google Scholar. The first five pages of records for each search were checked. Both searches were conducted on 26th March, 2020. The search strings were:

("defensive medicine" OR "defensive practice" OR "defensive practices" OR "defensive work" OR "defensive working") AND (qualitative OR interview OR interviews OR interviewed OR interviewing OR "focus group" OR "focus groups")

fear AND (legal OR liability OR complaint OR complaints OR litigation OR compensation OR damages OR "being sued" OR negligence OR regulation OR disciplinary OR discipline) AND (qualitative OR interview OR interviews OR "focus group" OR "focus groups")

The first string returned 27 potential includes in the first five pages of results. Thirteen were also identified by the database searches, eight were exclude 1, one was exclude 3, one was exclude 2, two were conference abstracts (one for a systematic review) – the author was emailed and replied that the data were not available at that time, one was in a foreign language, and one was a conference abstract linked to an included paper.

The second string returned seven potential includes in the first five pages. Three were in the database search results, two were exclude 1, one was exclude 3, and one was exclude 4.

Reference checking
The reference lists of all 15 included studies were checked. No new studies were identified. Most references were pre-2000 or already identified by the database searches. Across all studies, 16 references were exclude 1, one was exclude 2, 14 were exclude 3, one was exclude 4, and one was in a foreign language.
The references of three systematic reviews were checked, on 23rd March. From these, 35 references were considered to be potentially relevant; 21 were identified by the database searches, seven were exclude 3, and the remaining seven were exclude 1.
Websites
All website searches were conducted on 27th March, 2020. All sites were browsed for relevant research, as well as being searched as follows:
The General Medical Council (GMC) website at https://www.gmc-uk.org/ was searched using the term "defensive". This returned 15 items; all were exclude 1.
The Care Quality Commission (CQC) website at https://www.cqc.org.uk/ was searched using the term "defensive". Ten items were listed; none reported the views of clinicians on defensive medicine (all exclude 1).
The Professional Standards Authority (PSA) website at https://www.professionalstandards.org.uk/publications/research-papers was searched using the

term “defensive medicine”. This identified 11 articles; two were exclude 4, and the others were all exclude 1.
The Health and Care Professions Council (HCPC) https://www.hcpc-uk.org/resources/?Query=&Categories=48 was searched using the term “defensive medicine”. This identified one article, which was exclude 1.
The General Dental Council (GDC) website at https://www.gdc-uk.org/ was searched using the term “defensive”. This returned 23 results; all were exclude 1.

Forward citation searches

Web of Science Core Collection
Clarivate analytics https://clarivate.com/
6th April 2020
142 cites

1. Assing H, Lykkegaard J, Pedersen LB, Pedersen KM, Munck A, Andersen MK. How is defensive medicine understood and experienced in a primary care setting? A qualitative focus group study among Danish general practitioners. *BMJ Open* 2017;**7**:e019851.

- 7 cites

2. Assing H, Bjornskov P, Lykkegaard J, Moller P, Andersen MK. A colonized general practice? A critical habermasian analysis of how general practitioners experience defensive medicine in their everyday working life. *Health: an Interdisciplinary Journal for the Social Study of Health, Illness & Medicine* 2019:1363459319857461.

- not in Web of Science Core Collection

3. Bradder Annette M. *Reflexivity in professional practise and the social construction of defensive medicine: a study of discourses of risk in medical practice*. Ann Arbor; 2007.

- not in Web of Science Core Collection

4. Broom A, Kirby E, Gibson AF, Post JJ, Broom J. Myth, Manners, and Medical Ritual: Defensive Medicine and the Fetish of Antibiotics. *Qual Health Res* 2017;**27**:1994-2005.

- 13 cites

5. Cunningham W, Dovey S. Defensive changes in medical practice and the complaints process: a qualitative study of New Zealand doctors. *N Z Med J* 2006;**119**:U2283.

- 12 cites

6. Hammer R. Risk of Malpractice Claims and Changes in Professional Autonomy: A Qualitative Study of Obstetrician-Gynaecologists in Switzerland. *Swiss Journal of Sociology* 2017;**43**:163.

- not in Web of Science Core Collection

7. Hindley C, Thomson AM. Intrapartum fetal monitoring and the spectre of litigation: A qualitative study of midwives' views. *Clinical Governance* 2007;**12**:233-43.

– 10 cites

8. Hood L, Fenwick J, Butt J. A story of scrutiny and fear: Australian midwives' experiences of an external review of obstetric services, being involved with litigation and the impact on clinical practice. *Midwifery* 2010;**26**:268-85.

– 33 cites

9. Manuel J, Crowe M. Clinical responsibility, accountability, and risk aversion in mental health nursing: a descriptive, qualitative study. *International Journal of Mental Health Nursing* 2014;**23**:336-43.

– 22 cites

10. Papadopoulos Daphne A. *Obstetricians' reactions to the threat of medical malpractice litigation*. 2009.

– not in Web of Science Core Collection

11. Robertson JH, Thomson AM. An exploration of the effects of clinical negligence litigation on the practice of midwives in England: A phenomenological study. *Midwifery* 2016;**33**:55-63.

– 8 cites

12. Ruston A. Risk, anxiety and defensive action: general practitioner's referral decisions for women presenting with breast problems. *Health, Risk & Society* 2004;**6**:25-38.

– 7 cites

13. Spendlove Z. Risk and boundary work in contemporary maternity care: tensions and consequences. *Health, Risk and Society* 2018;**20**:63-80.

– 2 cites

14. Surtees R. 'Everybody expects the perfect baby...and perfect labour...and so you have to protect yourself ': discourses of defence in midwifery practice in Aotearoa/New Zealand. *Nurs Inq* 2010;**17**:82-92.

– 8 cites

15. Symon A. Obstetric litigation: effects on clinical practice. *Gynakol Geburtshilfliche Rundsch* 2000;**40**:165-71.

– 2 cites

16. Symon A. Litigation and changes in professional behaviour: a qualitative appraisal. *Midwifery* 2000;**16**:15-21.

– 10 cites

17. Wier JP. Protecting the public: an investigation of midwives perceptions of regulation and the regulator. *Midwifery* 2017;**52**:57-63.

- 0 cites

Description of Update Searches

Update searches were run by an Information Specialist (HF) between 12-17th October 2023, using all the databases from the original searches.

The update searches used identical search strategies except for: changes to date limits on CINAHL, ProQuest ASSIA, ProQuest Sociological Abstracts, and ProQuest Dissertations & Theses; and changes to subject headings on ProQuest ASSIA and ProQuest Sociological Abstracts where these needed to be updated. For ProQuest ASSIA, ProQuest Sociological Abstracts, and ProQuest Dissertations & Theses multiple searches were incorporated into several lines in order to run fewer search lines overall.

The results of the databases were deduplicated against each other in a separate EndNote 20 Library before being merged with the results of the original EndNote Library and deduplicated for a second time. This ensured that all new records retrieved by the search and not screened previously could be retrieved.
