# Supplementary material for: Defensive healthcare practice: systematic review of qualitative evidence
Source: BMJ Open. 2024 Jul 18;14(7):e085673. doi: 10.1136/bmjopen-2024-085673 (PMC11261683; doi:10.1136/bmjopen-2024-085673)
Supplement: online supplemental file 2 [file bmjopen-14-7-s002.pdf]

Defensive healthcare practice: Systematic review of qualitative evidence

Appendix B. References excluded at full text stage

Al-Balas QAE, Al-Balas HAE. The ethics of practicing defensive medicine in Jordan: a diagnostic study. *BMC Medical Ethics* 2021;22:87. doi: 10.1186/s12910-021-00658-8	EX 1
Alonso Perez d, Agreda, Febrel Bordeje M, et al. Factors related to inappropriate referral between primary and specialized care: qualitative study among primary care physicians. [Spanish] Factores asociados a la derivacion inadecuada entre atencion primaria y especializada: estudio cualitativo en medicos de atencion primaria. *Gaceta sanitaria / SESPAS* 2000;14(2):122-30.	EX 3
Bassett KL, Iyer N, Kazanjian A. Defensive medicine during hospital obstetrical care: a byproduct of the technological age. *Social Science & Medicine* 2000;51:523-37.	EX 1
Biancucci M. Les ajustements de la technique médicale face aux règles de droit. *Sociologie du travail* 2011;53(3):369-87.	EX 6
Bolton C, Goldsmith P. Complaints from patients with functional neurological disorders: a cross-sectional UK survey of why patients complain and the effect on the clinicians who look after them. *BMJ Open* 2018;8:e021573. doi: https://dx.doi.org/10.1136/bmjopen-2018-021573	EX 1
Bow JN, Gottlieb MC, Siegel JC, et al. Licensing board complaints in child custody practice. *Journal of Forensic Psychology Practice* 2010;10:403-18. doi: http://dx.doi.org/10.1080/15228932.2010.489851	EX 1
Broom J, Broom A, Adams K, et al. What prevents the intravenous to oral antibiotic switch? A qualitative study of hospital doctors' accounts of what influences their clinical practice. *Journal of Antimicrobial Chemotherapy* 2016;71:2295-99. doi: http://dx.doi.org/10.1093/jac/dkw129	EX 3
Calcaterra Susan L, Drabkin Anne D, Doyle R, et al. A Qualitative Study of Hospitalists' Perceptions of Patient Satisfaction Metrics on Pain Management. *Hospital Topics* 2017;95:18-26. doi: 10.1080/00185868.2017.1300479	EX 3
Canyon S. Between Professional Condescension and Chronic Victimhood: Medical Negligence Claims As Interpreted and Confronted by Israeli Physicians. *Social Issues in Israel* 2007;1:190-217.	EX 6
Catino M, Celotti S. Detection and prevention of adverse drug events: information technologies and human factors. The problem of defensive medicine: two Italian surveys. *Studies in Health Technology & Informatics* 2009;148:206-21.	EX 1
Charani E, Ahmad R, Rawson TM, et al. The Differences in Antibiotic Decision-making Between Acute Surgical and Acute Medical Teams: An Ethnographic Study of Culture and Team Dynamics. *Clinical Infectious Diseases* 2019;69:12-20. doi: https://dx.doi.org/10.1093/cid/ciy844	EX 3
Chard R. How perioperative nurses define, attribute causes of, and react to intraoperative nursing errors. *AORN Journal* 2010;91:132-45. doi: https://dx.doi.org/10.1016/j.aorn.2009.06.028	EX 1
Clare B, Paul G. Complaints from Patients with Functional Disorders. *Journal of Neurology, Neurosurgery and Psychiatry Conference: Association of British Neurologists Meeting, ABN* 2018;89 doi: 10.1136/jnnp-2018-ABN.134	EX 1

Colver Keith A. Ambulance Service Treat and Refer guidelines: a qualitative investigation into the use of Treat and Refer Guidelines by ambulance clinicians. *University of Stirling* 2012	EX 3
Cunningham W, Dovey S. The effect on medical practice of disciplinary complaints: potentially negative for patient care. *New Zealand Medical Journal* 2000;113:464-7.	EX 4
Dias MA, Deslandes SF. [Cesarean sections: risk perception and indication by attending obstetricians in a public maternity hospital in Rio de Janeiro]. *Cadernos de Saude Publica* 2004;20:109-16.	EX 3
Durupt M, Bouchy O, Christophe S, et al. Telemedicine in rural areas: general practitioners' representations and experiences. *Sante Publique (Vandoeuvre-Les-Nancey)* 2016;28:487-97.	EX 3
Elsom S, Happell B, Manias E. Exploring the expanded practice roles of community mental health nurses. *Issues in Mental Health Nursing* 2007;28:413-29.	EX 3
Figon S, Chaneliere M, Moreau A, et al. [Impact of adverse events on the practices of 15 general practitioners]. *Presse Medicale* 2008;37:1220-7. doi: https://dx.doi.org/10.1016/j.lpm.2007.09.029	EX 3
Fineberg AE, Tilton ZA. VBAC in the trenches: a community perspective. *Clinical Obstetrics & Gynecology* 2012;55:997-1004. doi: https://dx.doi.org/10.1097/GRF.0b013e31826fe5fa	EX 1
Frati P, Busardo FP, Sirignano P, et al. Does defensive medicine change the behaviors of vascular surgeons? A qualitative review. *BioMed Research International* 2015;2015:170692. doi: https://dx.doi.org/10.1155/2015/170692	EX 1
Fullen BM, Doody C, David B, et al. Chronic low back pain: non-clinical factors impacting on management by Irish doctors. *Irish Journal of Medical Science* 2008;177:257-63. doi: https://dx.doi.org/10.1007/s11845-008-0174-7	EX 3
Gabriel L, Reed R. P35 - We may be able to save more mothers and babies but what else are we risking?...Australian College of Midwives National Conference – Be the Change, September 12-14, 2023, Adelaide, South Australia. *Women & Birth* 2023;36:S44-S44. doi: 10.1016/j.wombi.2023.07.117	EX 3
Gott M, Gardiner C, Small N, et al. The effect of the Shipman murders on clinician attitudes to prescribing opiates for dyspnoea in end-stage chronic obstructive pulmonary disease in England. *Progress in Palliative Care* 2010;18:79-84. doi: http://dx.doi.org/10.1179/096992610X12624290276700	EX 4
Gruppetta E. Ethical issues for radiographers: general observations and a pilot qualitative study. *Radiation Protection Dosimetry* 2009;135:88-9. doi: https://dx.doi.org/10.1093/rpd/ncp103	EX 3
Guedes CRS, Leite ICG, Campos M, et al. Plain access to justice and the orthodontist's activity in Brazil: vulnerability in the professional practice in the face of risks of malpractice lawsuits. *Dental Press Journal of Orthodontics* 2018;23:88-93. doi: https://dx.doi.org/10.1590/2177-6709.23.4.088-093.sar	EX 2
Hahne J, Liang T, Khoshnood K, et al. Breaking bad news about cancer in China: Concerns and conflicts faced by doctors deciding whether to inform patients. *Patient Education & Counseling* 2019;17:17. doi: https://dx.doi.org/10.1016/j.pec.2019.08.022	EX 4
Hall MA, Peeples RA, Lord RW, et al. Liability implications of physician-directed care coordination. *Annals of Family Medicine* 2005;3:115-21.	EX 4
Hall MA. Employers' liability risk for managed care injuries. *Benefits Quarterly*	EX 2

2006;22:45-8.	
Hasan MDA, Shokry DA, Mahmoud RH, et al. Defensive Medicine Practice in Different Specialties among Junior Physicians in KasrAlAiny Hospitals, Egypt. *Indian Journal of Community Medicine* 2021;46:752-56. doi: 10.4103/ijcm.IJCM_143_21	EX 1
Hellyer P, Radford DR. An evaluation of defensive dentistry: w(h)ither the profession? *British Dental Journal* 2017;223:885-88. doi: https://dx.doi.org/10.1038/sj.bdj.2017.996	EX 1
Hindley C, Hinsliff SW, Thomson AM. English midwives' views and experiences of intrapartum fetal heart rate monitoring in women at low obstetric risk: conflicts and compromises. *Journal of Midwifery and Women's Health* 2006;51:354-60.	EX 3
Hoffman Bruce L. The Cultural Power of Law: The Criminalization, Organization, and Mobilization of Independent Midwifery, 2004.	EX 3
Jefford E, Jomeen J. "Midwifery Abdication": a finding from an interpretive study. *International Journal of Childbirth* 2015;5:116-25.	EX 3
Johnson CE, Bunderson JS. Enacting litigious environments: Litigation and Florida's nursing homes. *Health Care Management Review* 2002;27:7-20. doi: http://dx.doi.org/10.1097/00004010-200207000-00002	EX 1
Kirkham M, Stapleton H. The culture of the maternity services in Wales and England as a barrier to informed choice. *In: Kirkham M ed Informed choice in maternity care Basingstoke: Palgrave Macmillan* 2004:117-45.	EX 3
Kitch BT, Desroches C, Lesser C, et al. Systems model of physician professionalism in practice. *Journal of Evaluation in Clinical Practice* 2013;19:1-10. doi: https://dx.doi.org/10.1111/j.1365-2753.2011.01680.x	EX 3
Kucuk M. Defensive medicine among obstetricians and gynaecologists in Turkey. *Journal of Obstetrics & Gynaecology* 2018;38:200-05. doi: https://dx.doi.org/10.1080/01443615.2017.1340933	EX 1
Li S, Brantley E. Malpractice Liability Risk and Use of Diagnostic Imaging Services: A Systematic Review of the Literature. *Journal of the American College of Radiology* 2015;12:1403-12. doi: https://dx.doi.org/10.1016/j.jacr.2015.09.015	EX 1
Litchfield IJ, Lilford RJ, Bentham LM, et al. A qualitative exploration of the motives behind the decision to order a liver function test in primary care. *Quality in Primary Care* 2014;22:201-10.	EX 3
Litorp H, Mgaya A, Mbekenga C, et al. Fear, blame and transparency: Caregivers' rationales of a high caesarean section rate in a low-resource setting. *International Journal of Gynecology and Obstetrics* 2015;131:E536.	EX 3
Lockwood EB, Fealy GM. Nurse prescribing as an aspect of future role expansion: the views of Irish clinical nurse specialists. *Journal of Nursing Management* 2008;16:813-20. doi: https://dx.doi.org/10.1111/j.1365-2934.2008.00853.x	EX 1
Lundahl A, Helgesson G, Juth N. Psychiatrists' motives for practising in-patient compulsory care of patients with borderline personality disorder (BPD). *International Journal of Law & Psychiatry* 2018;58:63-71. doi: https://dx.doi.org/10.1016/j.ijlp.2018.03.005	EX 3
Lundquist M, Westin J. [Constant fear of liability reporting in physicians' everyday work. Impressions from interviews with physicians in Western Gotaland]. *Lakartidningen* 2003;100:3160-1.	EX 3
McGivern G, Fischer M. Medical regulation, spectacular transparency and the blame business. *Journal of Health Organization & Management* 2010;24:597-610.	EX 4

McGivern G, Fischer MD. Reactivity and reactions to regulatory transparency in medicine, psychotherapy and counselling. *Social Science & Medicine* 2012;74:289-96. doi: https://dx.doi.org/10.1016/j.socscimed.2011.09.035	EX 4
Mello MM, Kelly CN. Effects of a professional liability crisis on residents' practice decisions. *Obstetrics & Gynecology* 2005;105:1287-95.	EX 4
Mello MM, Studdert DM, DesRoches CM, et al. Caring for patients in a malpractice crisis: physician satisfaction and quality of care. *Health Affairs* 2004;23:42-53.	EX 3
Morata L. An evolutionary concept analysis of futility in health care. *Journal of Advanced Nursing* 2018;74:1289-300. doi: http://dx.doi.org/10.1111/jan.13526	EX 1
Moxey A, Robertson J, Newby D, et al. Computerized clinical decision support for prescribing: Provision does not guarantee uptake. *Journal of the American Medical Informatics Association* 2010;17:25-33. doi: http://dx.doi.org/10.1197/jamia.M3170	EX 3
Mulcahy L. From fear to fraternity: a socio-legal analysis of doctors' responses to being called to account by patients, 2000.	EX 4
Mullen R, Admiraal A, Trevena J. Defensive practice in mental health. *New Zealand Medical Journal* 2008;121:85-91.	EX 1
Munno A. A complaint that changed my practice: BMJ BMJ. *British Medical Journal* 2006;332:1092.	EX 1
Nash K. Better safe than sorry: Derms share views on practicing and teaching defensive medicine. *Dermatology Times* 2012;33:21-22.	EX 1
Neligan A, Long E, O'Connor M, et al. Irish Consultant's attitudes towards the climate of increasing medical litigation in Ireland [9]. *Irish Medical Journal* 2005;98:125.	EX 1
Nicholls JA, Potts HW, Coleman B, et al. Legal and professional implications of shared care: a case study in oral anticoagulation stroke prevention therapy. *BMC Health Services Research* 2015;15:93. doi: https://dx.doi.org/10.1186/s12913-015-0756-9	EX 1
Oldenburg D, Wagner HO, Steinhauser J. Legal Implications of Physician Action - Dealing Professionally with Treatment Errors. [German] Juristische Implikationen arztlichen Handelns - Professioneller Umgang mit Behandlungsfehlern. *Zeitschrift fur Allgemeinmedizin* 2022;98(12):441-45. doi: 10.53180/zfa.2022.0441-0445	EX 1
Oliveira AS, Guerreiro MP. 'Everyone plays defence': a qualitative exploration of issues that influence the prescribing of antibiotics by Portuguese dentists. *Drugs and Therapy Perspectives* 2017;33:234-40. doi: http://dx.doi.org/10.1007/s40267-017-0388-8	EX 3
Osorio D, Ribera A, Solans-Domenech M, et al. Healthcare professionals' opinions, barriers and facilitators towards low-value clinical practices in the hospital setting. *Gaceta Sanitaria* 2019;10:10. doi: https://dx.doi.org/10.1016/j.gaceta.2018.11.007	EX 3
Pugh D. The phoenix process: a substantive theory about allegations of unprofessional conduct. *Journal of Advanced Nursing* 2009;65:2027-37. doi: https://dx.doi.org/10.1111/j.1365-2648.2009.05038.x	EX 4
Renkema E, Broekhuis M, Ahaus K. Conditions that influence the impact of malpractice litigation risk on physicians' behavior regarding patient safety. *BMC Health Services Research* 2014;14:38. doi: https://dx.doi.org/10.1186/1472-6963-14-38	EX 4
Riascos Aldemar O, Florez Yohana L. Defensive practices among healthcare workers: An overview of the scientific literature. 2021	EX 1
Ries N, Hacker C, McCaffery K, et al. A mapping review of empirical research on defensive practice. *BMJ Evidence-Based Medicine* 2019;24(Supplement 2):A32. doi:	EX 1

10.1136/bmjebm-2019-POD.67	
Ries N, Hacker C, McCaffery K, et al. Medico-legal experts' views on psychosocial drivers of defensive practice: A qualitative interview study. *BMJ Evidence-Based Medicine* 2019;24(Supplement 2):A48-A49. doi: 10.1136/bmjebm-2019-POD.99	EX 1
Riotta S, Bruccoleri M. Revisiting the patient-physician relationship under the lens of value co-creation and defensive medicine. *Journal of Service Theory and Practice* 2021;31:868-92. doi: 10.1108/JSTP-06-2020-0142	EX 4
Robertson Judith H, Thomson Ann M. A phenomenological study of the effects of clinical negligence litigation on midwives in England: The personal perspective. *Midwifery* 2014;30:e121-30. doi: 10.1016/j.midw.2013.12.003	EX 4
Romøren M, Pedersen R, Førde R. How do nursing home doctors involve patients and next of kin in end-of-life decisions? A qualitative study from Norway. *BMC Medical Ethics* 2016;17:1-8. doi: 10.1186/s12910-016-0088-2	EX 3
Savage W, Francome C. British consultants' attitudes to caesareans. *Journal of Obstetrics & Gynaecology* 2007;27:354-9.	EX 3
Scamell M. The fear factor of risk -- clinical governance and midwifery talk and practice in the UK. *Midwifery* 2016;38:14-20. doi: 10.1016/j.midw.2016.02.010	EX 4
Schwarz JKK. Assistance in dying: the nurse's experience, 2002.	EX 3
Seger T, Harpaz I, Meshulam I. Israeli physicians manage risk of litigation: Predicting empowerment role model. *The International Journal of Human Resource Management* 2011;22:2442-62. doi: http://dx.doi.org/10.1080/09585192.2011.584408	EX 1
Smith-Oka V. Cutting Women: Unnecessary cesareans as iatrogenesis and obstetric violence. *Social Science & Medicine* 2022;296:114734. doi: 10.1016/j.socscimed.2022.114734	EX 3
Stahlke W. The impact of regulatory perspectives and practices on professional innovation in nursing. *Nursing Inquiry* 2018;25:01. doi: https://dx.doi.org/10.1111/nin.12212	EX 3
Sugiyama S, Asakura K, Takada N. Japanese nurse practitioners' legal liability ambiguity regarding their medical practice: a qualitative study. *BMC Nursing* 2020;19:62. doi: 10.1186/s12912-020-00458-2	EX 4
Suresh L. Factors Influencing Doctors Ordering of Clinical Lab Tests: A Qualitative Study, 2017.	EX 3
Ünal Ö, Akbolat M, Amarat M. Determination of the Physician's Experience of the Practices, Antecedents and Outcomes of Defensive Medicine Practices. *Journal of Health Management* 2023;25:254-62. doi: 10.1177/09720634231175741	EX 1
Ünal Ö. Defansif Tıp Uygulamaları,Öncülleri ve Sonuçları, 2020.	EX 6
Velázquez T, Laura E. Influencia de los medios de comunicación sobre la práctica médica defensiva: un abordaje cualitativo desde la perspectiva de médicos. *Revista Espanola de Comunicacion en Salud* 2020;11:178-92. doi: 10.20318/recs.2020/5141	EX 6
Velázquez TLE. From the right to health to defensive medicine: perspective of physicians in the care of patients with type II diabetes [Del derecho a la salud a la medicina defensiva: perspectiva de médicos en la atención a pacientes con diabetes tipo II]. *Rev CONAMED 2019* 2019;24(2):64-72.	EX 6
Wahlberg A, Hogberg U, Emmelin M. The erratic pathway to regaining a professional self-image after an obstetric work-related trauma: A grounded theory study. *International Journal of Nursing Studies* 2019;89:53-61. doi:	EX 4

https://dx.doi.org/10.1016/j.ijnurstu.2018.07.016	
Weaver JJ. Thoughts on caesarean section. *MIDIRS Midwifery Digest* 2001;11:S16-S18.	EX 4
Wick MR, Foucar E, Allen PW, et al. Medicolegal liability in pathology: an international perspective. *Seminars in Diagnostic Pathology* 2007;24:65-76.	EX 1
Zheng J, Yang F, Li W, et al. Prevalence and determinants of defensive medicine among physicians: a systematic review and meta-analysis. 2023	EX 1
