# Supplementary material for: Defensive healthcare practice: systematic review of qualitative evidence
Source: BMJ Open. 2024 Jul 18;14(7):e085673. doi: 10.1136/bmjopen-2024-085673 (PMC11261683; doi:10.1136/bmjopen-2024-085673)
Supplement: online supplemental file 3 [file bmjopen-14-7-s003.pdf]

Defensive healthcare practice: Systematic review of qualitative evidence

Appendix C. Results of quality assessment

First author	Abstract and title	Introduction and aims	Method and data	Sampling	Data analysis	Ethics and bias	Results	Transferability or generalizability	Implications and usefulness
Assing Hvidt et al. [21,22]	Good	Good	Good	Fair	Fair	Fair	Good	Fair	Good
Bradder [23]	Poor	Good	Good	Fair	Fair	Good	Fair	Poor	Poor
Broom et al. [24]	Fair	Good	Fair	Poor	Fair	Fair	Good	Poor	Poor
Cunningham & Dovey [25]	Good	Good	Fair	Fair	Fair	Fair	Fair	Poor	Fair
Eftekhari et al. [26]	Good	Good	Good	Poor	Fair	Fair	Fair	Poor	Fair
Hammer [27]	Fair	Good	Fair	Poor	Fair	Fair	Good	Poor	Fair
Hindley & Thomson [28]	Good	Good	Fair	Poor	Good	Fair	Good	Poor	Good
Hood et al. [29]	Good	Good	Good	Fair	Fair	Good	Good	Fair	Fair
Manuel & Crowe [30]	Good	Good	Good	Poor	Good	Good	Fair	Poor	Fair
Papadopoulos [31]	Good	Good	Good	Poor	Fair	Fair	Fair	Poor	Good
Ries et al. [32]	Good	Good	Good	Poor	Fair	Fair	Good	Poor	Fair
Robertson & Thomson [33]	Good	Good	Fair	Fair	Good	Good	Good	Poor	Good
Ruston [34]	Good	Good	Good	Good	Fair	Fair	Fair	Poor	Fair
Spendlove [35]	Fair	Good	Fair	Poor	Fair	Good	Good	Poor	Poor
Surtees [36]	Fair	Fair	Very poor	Very poor	Very poor	Poor	Fair	Very poor	Fair
Symon [37,38]	Good	Fair	Fair	Poor	Fair	Fair	Fair	Poor	Fair
Wier [39]	Good	Good	Fair	Poor	Good	Good	Fair	Poor	Fair
