# Supplementary material for: Defensive healthcare practice: systematic review of qualitative evidence
Source: BMJ Open. 2024 Jul 18;14(7):e085673. doi: 10.1136/bmjopen-2024-085673 (PMC11261683; doi:10.1136/bmjopen-2024-085673)
Supplement: Supplementary file 4 [file bmjopen-2024-085673.reviewer_comments.pdf]

PEER REVIEW HISTORY

BMJ Open publishes all reviews undertaken for accepted manuscripts. Reviewers are asked to complete a checklist review form (**http://bmjopen.bmj.com/site/about/resources/checklist.pdf**) and are provided with free text boxes to elaborate on their assessment. These free text comments are reproduced below.

ARTICLE DETAILS

**TITLE (PROVISIONAL)**	Defensive healthcare practice: Systematic review of qualitative evidence
**AUTHORS**	Lorenc, Theo; Khouja, Claire; Harden, Melissa; Fulbright, Helen; Thomas, James

VERSION 1 – REVIEW

**REVIEWER**	Fantus, Sophia<br>The University of Texas at Arlington
**REVIEW RETURNED**	18-Mar-2024

**GENERAL COMMENTS**	Thank you for allowing me the time to review this interesting manuscript. I think the practice of defensive medicine is both timely and relevant. There are a number of ways in which the authors could strengthen this paper:<br>1. I would have liked to see a much richer and more in-depth introduction. The authors spend a considerable of time discussing the definition and operationalization of defensive medicine but rather they concentrate their research questions on defensive practices and procedures as well. It may be helpful to divide the introduction into various sub-themes that create a narrative as to why this qualitative systematic review is important and, in doing so, perhaps lay the foundation as to why the authors selected a qualitative review rather than both quantitative and qualitative empirical work. I think the authors should not specific findings of other studies and perhaps also contextualize the study. The authors are perhaps right that most of the information pertains to the U.S. but that might be because of the way in which the healhcare system and litigation system is set up in the U.S. The authors may be correct that you cannot generalize to other countries but then the readers do not have a good vantage point in which to contextualize the findings (specifically those that may come from the UK and New Zealand). Therefore, at some point in the article, the authors need to contextualize the findings and write in some information pertaining to the healthcare system in these countries or rather keep the review to the U.S. and place a subsection that contextualizes the U.S. Without this, the authors may fall into the same trap of not having contextualized defensive medicine themselves.<br>2. There needs to be much more information on the methods section in terms of the rationale for all decisions made throughout the review and a much richer section on how the team analyzed the findings and how they came to the themes through qualitative analysis. It may help to have a table that has the themes prevalent in each incorporated manuscript. As of right now, how the authors reviewed the thematic analysis from the original manuscripts to their analysis is not quite clear. Clarifying this process would help

	readers make sense of the research question. As well as rationalizing why only the perspective of clinicians were included and if there are any studies on patient perspectives (e.g., do patients also view decisions made by clinicians as rooted in defensive practice). If this is beyond the scope, this should be articulated.<br>3. There are places in the manuscript where it says in bold (Error! Reference source not found.). Please fix. For example, page 6, line 9 and page 6, line 17.<br>4. The results are really interesting. It would be helpful to break down these results into sub-themes and topics. The way it reads is very broad scoped and some of the quotations do not align with the topic chosen. That way, it is unclear how the authors analyzed the results as these seem to be just topics or categories and not necessarily themes that arose. I think the manuscript would benefit from some in-depth analysis regarding the topics that are explored to really bring forward an answer to their research question. Especially as they are looking at qualitative studies, it is important to really understand the in-depth rich perspectives voiced in these manuscripts and also the themes that arose in these studies - what new information are the authors bringing and how are they synthesizing these results? The authors may want to look at some studies that utilize a QIMS.<br>5. The authors concentrate a lot on C-sections and midwives and it may be relevant to have some conclusion/discussion on why this may be an important research topic in scholarship, especially within the U.S.<br>6. There is no analysis in terms of discrepancies in geographical context. Did the authors see any differences/disparities between qualitative findings, for example, in Iran vs New Zealand vs England? The authors discuss at the beginning ways to generalize and the importance of including a global perspective but it is also incredibly important to understand these various contexts and how defensive medicine may be understood or examined differently or even practiced differently - there may be differences with respect to administrative hurdles but there may also be differences to social context in terms of the way physicians are valued or processes surrounding childbirth and prenatal care. There ought to be some level of analysis that describes these variances. In doing so, the table that has the information, it may be helpful to have percentage of clinicians when it says "doctors" - any doctors? family doctors? is there information in these studies on the division of fields of specialty? There is just a lot of information to disentangle and I think to do this study justice is to examine some of that sociopolitical contexts of places included.<br><br>This was a really interesting manuscript, and the practice of defensive medicine has implications for both clinicians and patients, which ought to be considered in the discussion (especially when patients wind up having to pay for these choices or dealing with insurance). There are also some grammar and spelling errors. Please review.<br><br>Thank you again.

**REVIEWER**	Yang, Fan<br>Shanghai Jiao Tong University
**REVIEW RETURNED**	27-Apr-2024

GENERAL COMMENTS	I have following comments for the further improvement of this manuscript:<br>1. The current title is vague and cannot reflect the research questions of the paper<br>2. Please justify why only review qualitative evidence. A lot of research has been done on this topic using quantitative methods.<br>3. Significant differences could exist between physicians from different specialities and with different practicing years. It's a pity for not discussing these influencing factors in this review.

REVIEWER	Yang, Fan<br>Shanghai Jiao Tong University
REVIEW RETURNED	27-Apr-2024

GENERAL COMMENTS	I have following comments for the further improvement of this manuscript:<br>1. The current title is vague and cannot reflect the research questions of the paper<br>2. Please justify why only review qualitative evidence. A lot of research has been done on this topic using quantitative methods.<br>3. Significant differences could exist between physicians from different specialities and with different practicing years. It's a pity for not discussing these influencing factors in this review.

VERSION 1 – AUTHOR RESPONSE

	R1	
1	I would have liked to see a much richer and more in-depth introduction. The authors spend a considerable of time discussing the definition and operationalization of defensive medicine but rather they concentrate their research questions on defensive practices and procedures as well. It may be helpful to divide the introduction into various sub-themes that create a narrative as to why this qualitative systematic review is important and, in doing so, perhaps lay the foundation as to why the authors selected a qualitative review rather than both quantitative and qualitative empirical work. I think the authors should not specific findings of other studies and perhaps also contextualize the study. The authors are perhaps right that most of the information pertains to the U.S. but that might be because of the way in which the healhcare system and litigation system is set up in the U.S. The authors may be correct that you cannot generalize to other countries but then the readers do not have a good vantage point in which to contextualize the findings (specifically those that may come from the UK and New Zealand). Therefore, at some point in the article, the authors need to contextualize the findings and write in some information pertaining to the healthcare system in these countries or rather keep the review to the U.S. and place a subsection that contextualizes the U.S. Without this, the authors may fall into the same trap of not having contextualized defensive medicine themselves.	We have rewritten the introduction, adding material and new references to (1) bring out the need for a review of qualitative evidence and (2) expand on the points (which were admittedly very brief and vague in the original draft) about differences between countries to contextualise the findings as the reviewer suggests.<br>Added: “Defensive practice may have negative consequences for patient care, both directly due to the impacts of inappropriate treatment, and indirectly by undermining care quality (e.g. antibiotic stewardship)[2] and fostering unrealistic norms of clinical practice (so-called ‘gold plating’, where practices adopted defensively become incorporated into routine care).[3,4] Defensive practice has also widely been seen as a potential driver of excess healthcare costs, due to the additional costs of carrying out tests or procedures. […]Qualitative research may help to fill in the picture outlined by the quantitative data, by situating concerns about litigation or other relevant factors in the day-to-day realities of clinical practice. […]. The market for malpractice insurance in the USA, where many clinicians purchase

		cover as individuals, is quite different to that in other settings such as the UK where this is less common.[15] Concern about increased malpractice litigation and spiralling insurance costs, which have formed the background of thinking around defensive medicine in the USA,[16–18] have been less of a focus in other countries, and concerns about defensive medicine in the latter may relate to broader consequences such as patient complaints.[8] Hence, while self-reported rates of defensive practice appear to be high across countries, it is unclear how far the overarching theoretical narrative developed from the US data can be applied more generally. The broader context of clinicians' work – the incentive structures provided by different reimbursement systems, or the cultural norms which influence patients' expectations –also vary internationally in ways which may have an impact on defensive decision-making."
2	There needs to be much more information on the methods section in terms of the rationale for all decisions made throughout the review and a much richer section on how the team analyzed the findings and how they came to the themes through qualitative analysis. It may help to have a table that has the themes prevalent in each incorporated manuscript. As of right now, how the authors reviewed the thematic analysis from the original manuscripts to their analysis is not quite clear. Clarifying this process would help readers make sense of the research question. As well as rationalizing why only the perspective of clinicians were included and if there are any studies on patient perspectives (e.g., do patients also view decisions made by clinicians as rooted in defensive practice). If this is beyond the scope, this should be articulated.	We have added more detail under method, particularly around the synthesis. Added: "This tool provides a structured instrument to evaluate quality across several methodological domains, including sampling, data collection and analysis, ethics and bias, and transferability. Studies were not excluded nor downgraded based on quality assessment rating, but information on study quality was used informally to guide the synthesis […] The structure of the themes is shown in Table 1. The overarching framework consisted of three domains: definitions and understandings of defensive practice; causal factors influencing defensive practice; and the impacts of defensive practice across a range of domains. More specific codes were developed within this structure as shown in Table 1. Coding was inductive, with new codes developed in discussion between the two reviewers undertaking the analysis; where new codes were added, all data were reread to identify data corresponding to the code. Development of the coding structure was guided by thematic saturation." (see also Table 1).<br>The limitation to studies about clinicians was part of the scope, but we do recognise that this is a potential issue which needs more attention than it received in the draft. Added: "We

		focused on studies of clinicians (rather than patients or other stakeholders) partly for pragmatic reasons, in order to make the review manageable, and partly because we anticipated that patients would be less likely to frame views about care in terms of the impact of litigation (we return to this point as a potential limitation in the discussion below)."
3	There are places in the manuscript where it says in bold (Error! Reference source not found.). Please fix. For example, page 6, line 9 and page 6, line 17.	Our apologies for the technical error. This should now be fixed.
4	The results are really interesting. It would be helpful to break down these results into sub-themes and topics. The way it reads is very broad scoped and some of the quotations do not align with the topic chosen. That way, it is unclear how the authors analyzed the results as these seem to be just topics or categories and not necessarily themes that arose. I think the manuscript would benefit from some in-depth analysis regarding the topics that are explored to really bring forward an answer to their research question. Especially as they are looking at qualitative studies, it is important to really understand the in-depth rich perspectives voiced in these manuscripts and also the themes that arose in these studies - what new information are the authors bringing and how are they synthesizing these results? The authors may want to look at some studies that utilize a QIMS.	We have added more structure to the results section as requested (this should additionally help to address point 2 above). We have also revised the whole results section, bringing in more material from the original analysis to deepen the engagement with the themes as the reviewer suggests (the full text of all the additions is not reproduced here but is highlighted in the draft).
5	The authors concentrate a lot on C-sections and midwives and it may be relevant to have some conclusion/discussion on why this may be an important research topic in scholarship, especially within the U.S.	Added: "The large body of data on Caesarean sections and electronic foetal monitoring suggests that litigation risk may play an outsized role in obstetrics and midwifery; this may be due to the potentially severe and lifelong consequences of medical error in childbirth, and hence the potential for large compensation awards following litigation."
6	. There is no analysis in terms of discrepancies in geographical context. Did the authors see any differences/disparities between qualitative findings, for example, in Iran vs New Zealand vs England? The authors discuss at the beginning ways to generalize and the importance of including a global perspective but it is also incredibly important to understand these various contexts and how defensive medicine may be understood or examined differently or even practiced differently - there may be differences with respect to administrative hurdles but there may also be differences to social context in terms of the way physicians are valued or processes surrounding childbirth and prenatal care. There ought to be some level of analysis that describes these variances. In doing so, the table that has the information, it may be helpful to have percentage of clinicians when it says "doctors" - any doctors?	We have returned to the data to draw out international comparisons, but have been able to draw limited robust conclusions. Partly this is due to the relatively small amount of data from some countries – for example, there are some specific findings in the Iranian study which may relate to national differences, but it is difficult to draw conclusions about this from a single study. We have added the following under results: "Relatively few participants identified specific aspects of the legislative or policy context as potential drivers of defensive practice. In two studies, one from Denmark[24] and one from Australia[27] participants argued that private health insurance may be a motivator. In one study from

	family doctors? is there information in these studies on the division of fields of specialty? There is just a lot of information to disentangle and I think to do this study justice is to examine some of that sociopolitical contexts of places included.	Switzerland, participants identified lower levels of compensation awards and regulation on legal fees as limiting clinicians' litigation risk, and hence reducing defensive practice.[30] Some participants saw litigation risk as primarily a problem with the US healthcare system, and defensive practice as a symptom of "Americanisation"[30,39] (as we only located one study from the USA, which presented fairly limited relevant data, we cannot directly compare perceptions)."<br>In Table 1, the clinicians' specialisms correspond to the 'setting' column (where the setting is listed as 'hospital' or 'various' there is generally no further information on clinician specialisms). The main issue here in terms of the results is the specific status of obstetrics/midwifery – see response to point (5).
7	This was a really interesting manuscript, and the practice of defensive medicine has implications for both clinicians and patients, which ought to be considered in the discussion (especially when patients wind up having to pay for these choices or dealing with insurance). There are also some grammar and spelling errors. Please review.	We have reviewed the manuscript and removed any errors (particularly checking consistency in verb tenses).
	R2	
8	The current title is vague and cannot reflect the research questions of the paper	The research question was broadly defined as including any views data about defensive practice. Making the title more specific would be an inaccurate representation of the scope of the review.
9	Please justify why only review qualitative evidence. A lot of research has been done on this topic using quantitative methods.	R1 also made this point – see the comments in response to (1) above.
10	Significant differences could exist between physicians from different specialities and with different practicing years. It's a pity for not discussing these influencing factors in this review.	As regards specialties, see response to (6) above. As regards practicing years, we have revisited the analysis and added the following: "This may sometimes reflect a lack of confidence or experience among junior staff (or at least this perception by senior staff),[26] although participants in one study suggested that newly qualified clinicians were less likely to practice defensively as they had not had experience of litigation or adverse outcomes.[32]"
